# Re-designing irrigated intensive cereal systems through bundling precision agronomic innovations for transitioning towards agricultural sustainability in North-West India

**DOI:** 10.1038/s41598-019-54086-1

**Published:** 2019-11-29

**Authors:** H. S. Jat, P. C. Sharma, Ashim Datta, Madhu Choudhary, S. K. Kakraliya, Yadvinder-Singh, Harminder S. Sidhu, B. Gerard, M. L. Jat

**Affiliations:** 1 ICAR-Central Soil Salinity Research Institute (CSSRI), Karnal, Haryana India; 2 International Maize and Wheat Improvement Center (CIMMYT), New Delhi, India; 3 Borlaug Institute for South Asia (BISA)-CIMMYT, Ludhiana, India; 4 International Maize and Wheat Improvement Center (CIMMYT), El-Batan, Texcoco, Mexico

**Keywords:** Agroecology, Sustainability

## Abstract

A study was conducted to design productive, profitable, irrigation water¸ nitrogen and energy use efficient intensive cereal systems (rice-wheat; RW and maize-wheat; MW) in North-West India. Bundling of conservation agriculture (CA) with sub-surface drip irrigation termed as CA^+^ were compared with CA alone and conventional tillage based and flood irrigated RW rotation (farmer’s practice; ScI). In contrast to conventional till RW rotation which consumed 1889 mm ha^−1^ irrigation water (2-yr mean), CA^+^ system saved 58.4 and 95.5% irrigation water in RW and MW rotations, respectively. CA^+^ practices saved 45.8 and 22.7% of irrigation water in rice and maize, respectively compared to CA with flood irrigation. On a system basis, CA^+^ practices saved 46.7 and 44.7% irrigation water under RW (ScV) and MW (ScVI) systems compared to their respective CA-based systems with flood irrigation (ScIII and ScIV). CA^+^ in RW system recorded 11.2% higher crop productivity and improved irrigation water productivity by 145% and profitability by 29.2% compared to farmers’ practice. Substitution of rice with maize (MW system; ScVI) recorded 19.7% higher productivity, saved 84.5% of irrigation water and increased net returns by 48.9% compared to farmer’s practice. CA^+^ RW and MW system improved energy productivity by 75 and 169% and partial factor productivity of N by 44.6 and 49.6%, respectively compared to ScI. The sub-surface drip irrigation system saved the fertilizer N by 20% under CA systems. CA^+^ in RW and MW systems recorded ~13 and 5% (2-yr mean) higher profitability with 80% subsidy on installing sub-surface drip irrigation system and similar profitability without subsidy scenario compared with their respective flood irrigated CA-based systems.

## Introduction

Billions of people from South Asia depends on rice-wheat (RW) system (13.5 M ha) for their food security and livelihood^1^. Whereas, maize-wheat (MW) rotation is third most important cropping system (~1.86 M ha)^2^ and has potential to expand in view of emerging water crisis in the Indo-Gangetic plains. Over time, the sustainability of the intensive rice-wheat systems of North-West (NW) India has become a major challenge owing to faster depletion of groundwater table, stagnating or declining productivity growth, degrading soil health and environmental quality, and diminishing farm profitability^3,4^. Furthermore, projected climate change will increase future water demand for irrigation by ~10% with each °C rise in temperature whereas the availability will decline in the areas where irrigation is most needed^5^. On the other hand, increasing demands on water resources by increasing population and urbanization have reduced the water availability in South Asia^6^. Therefore, by 2050, India would face severe water constrains for both agriculture and domestic use^7^.

Rice is a water guzzling crop and consumes significant amount (about 50%) of total irrigation water use in Asia and it accounts for about 24–30% of the world total freshwater withdrawal^8^. During 2008–2012, the total fresh water withdrawals in India were about 761 billion m^3^ of which about 90% was used for agriculture^9^. Since the early 1970s (Green Revolution era), there has been a steady decline in groundwater table in most of the RW domain area of North-West (NW) India^6,10^. The decline in ground water table in NW India between 1973 and 2001 was about 0.2 m yr^−1^ which has accelerated by five-fold (1.0 m yr^−1^) between 2000 and 2006. This has also led to increased energy demand for pumping and increased costs for installing deep submersible pumps. Conventional cultivation practices of RW and MW system consumes 2150–2300 mm and 250–350 mm irrigation water for crop production, respectively^3,4^. If sustainable measures are not taken soon to ensure sustainable use of groundwater, the IGP of NW India may soon experience reduction of crop productivity and farm profitability, and shortages of potable water leading to extensive socioeconomic stresses.

In the conventional till (CT) RW system, repeated tillage and crop residue burning are the two other major causes of concern for soil health deterioration and environmental pollution; the key indicators of sustainability. Conservation agriculture (CA) based innovative agronomic management practices like zero-tillage (ZT)/No-tillage, crop establishment (smart seeding system/dry seeded rice), residue recycling, precision water and nutrient management etc. have been used as an alternative to conventional puddled transplanted rice (PTR) and CT wheat to improve farm productivity and farmers’ profitability^1,3^. Looking to the constraints of water shortages in future, it is imperative that we put more focus on re-designing intensive cereal systems through developing efficient and remunerative practices for increasing water productivity and farmer’s profitability in irrigated cereal systems in NW India^6^.

For precise water management, drip irrigation has shown several benefits in terms of irrigation water savings, increases in yield and quality, and increase in nutrient use efficiency in horticulture and vegetable crops^11^. To address water scarcity, surface drip irrigation has also been found a viable option for the cereal crops like maize^12,13^, rice and wheat^14,15^. However, adoption of surface drip irrigation in cereal systems has always remained cumbersome process of anchoring laterals at the beginning and removing after every crop due to field operations during the year under both conventional and conservation agriculture management systems. To address this bottleneck and improve farmers ease for acceptance of drip irrigation in cereal systems, subsurface drip irrigation (SDI) is a way forward. Unlike surface drip irrigation, SDI system limits evaporation loss from the soil surface, allows water and nutrients directly to the root zone that leads to efficient water use, prevents weed emergence, reduces labor cost, and allows direct seeding with no-tillage practices^12^. Fertigation through drip irrigation system reduces the N- volatilization and leaching losses thereby improving the nutrient use efficiency^16^. CA-based annual cereal rotations (RW and MW) allow crop residue recycling, saves irrigation water by reducing evaporative losses, lowers energy use by reducing the use of machinery for tillage and pumping, and increases farmers’ profit^4^.

Recently, Sidhu *et al*.^17^ have standardized the SDI system for CA-based RW and reported significant improvements in irrigation water and N use efficiency. However, information on the SDI system for the CA based RW and MW cropping systems is very limited under different soil and climate conditions from NW India as well as from other parts of South Asia and the Globe. The premise of our study is that adoption of CA-based new agronomic management practices with precision irrigation management (SDI) termed as CA^+^ and replacing rice with maize (low water requiring crop) and integration of short duration legumes (mungbean; *Vigna radiata*) may help achieving not only to achieve sustainable crop production in the IGP of NW India but also contributing to several sustainable development goals (SDGs). We hypothesized that bundling CA practices with SDI (CA^+^) will allow increased system productivity and farm profitability while improving the irrigation water productivity through significant saving in water use and improving yields in cereal based systems. The objective of our study was to design intensive cereal systems through bundling CA and SDI (CA^+^) for improving crop yields, water productivity, N and energy use efficiency, and farm profitability in NW India for a sustainable farming future.

## Results

### Weather conditions

The rainfall during both the years (2016–17 and 2017–18) was in accordance with the long-term annual average of 670 mm. During rice and maize growing periods, a total rainfall of 433 and 567 mm was received during the year 2016 and 2017, respectively. Rainfall distribution pattern was quite different in each season, with high (>200 mm) rainfall in August, 2016, and in June and September in 2017. Monthly pan evaporation (PE) pattern was nearly uniform across the rice/maize season but it was lowest in December in wheat growing season during both the years. The highest PE was recorded in the month of June, 2016 (234.3 mm) and in May 2017 (239.70 mm). In wheat growing season, almost similar amount of rainfall (188 mm in 2016–17 and 170 mm in 2017–18) was received during both the years. The rainfall was received in the month of January in 2016–17 while, from December to February in 2017–18. Monthly PE and temperature patterns during wheat growing season were more or less similar in both the years.

### Crop productivity

In the first year (2016–17), significantly lower rice equivalent yield (REY) (5.88–6.21 Mg ha^−1^) was recorded in scenario III (ScIII) and ScV, compared with other scenarios (Table 1). Highest REY of 7.61 Mg ha^−1^ under ScVI, which was at par with Sc1 and ScIV (Table 1). However, in second year, the REY was significantly lower in ScI, ScIII and ScV compared with other scenarios. There was no significant effect of SDI (CA^+^) on grain yields of rice and maize compared to the corresponding CA treatments. On average basis, 16.7% lesser yield of DSR was recorded with CA (ScIII), whereas with CA^+^ (ScV), the yield was improved over DSR with flood irrigation yet it was 10.6% lower compared to ScI (farmer’s practice). In wheat, 13.3% (2-yr mean) higher grain yield was recorded with CA (ScIII) compared with 5.67 Mg ha^−1^ in CT (ScI) (Table 1). Mean wheat yield was 17.0 and 10.8% higher under CA^+^ and CA-based cereal systems compared with CT, respectively. However, there was no significant effect of SDI on grain yield of wheat under CA-based wheat cultivation. Mungbean grain yield in ScII was 54% (2-yr mean) higher over the other scenarios whereas it was at par with under CA and CA^+^ scenarios (Table 1).

**Table 1:** Effect of management practices portfolios on grain yield, cost of cultivation with (80%) and without subsidy on SDI system and net returns under different scenarios during year 2016–17 and 2017–18.

Scenarios^a^	Grain yield (Mg ha^−1^)				Cost of cultivation (USD ha^−1^)				Net return (USD ha^−1^)			
	Rice equivalent	Wheat	Mungbean	System	Rice/Maize	Wheat	Mungbean	System	Rice/Maize	Wheat	Mungbean	System
*2016–17*												
ScI	7.51^Ab^	5.47^C^	-NA^c^ -	13.40^B^	687^A^	672^A^	-NA-	1359^A^	979^B^	828^C^	-NA	1807^C^
ScII	7.39^B^	5.72^BC^	0.50^A^	15.08^A^	655^A^	531^B^	150^A^	1335^A^	984^B^	1021^BC^	219^A^	2225^B^
ScIII	5.88^C^	6.35^AB^	0.15^B^	13.19^B^	600^B^	529^BC^	117^B^	1247^B^	705^C^	1225^AB^	−5^B^	1925^C^
ScIV	7.12^AB^ (7.66)*	6.53^AB^	0.15^B^	14.62^A^	568^BC^	527^BCD^	117^B^	1212^C^	1243^A^	1243^A^	−5 ^B^	2482^A^
ScV	6.21^C^	6.79^A^	0.20^B^	14.12^AB^	526^D^ (642)	525^CD^ (640)	114^B^	1165^D^ (1395)	852^BC^ (736)	1302 ^A^ (1186)	30^B^	2184^B^ (1953)
ScVI	7.61 ^A^ (8.20)	6.38^AB^	0.22^B^	15.14^A^	558^CD^ (674)	524^D^ (639)	114^B^	1196^CD^ (1426)	1289 ^A^ (1173)	1214^AB^ (1198)	45^B^	2547 ^A^ (2317)
*2017–18*												
ScI	6.57^C^	5.88^C^	-NA-	13.33^C^	703^B^	682^A^	-NA-	1385^B^	795^B^	1147^C^	-NA-	1941^D^
ScII	6.85^BC^	6.06^BC^	0.74^A^	16.19^AB^	656^C^	536^B^	227^A^	1418^A^	905^B^	1346^B^	315^A^	2567^BC^
ScIII	5.85^C^	6.58^AB^	0.45^B^	14.86^BC^	623^D^	532^B^	151^B^	1306^C^	710^B^	1490^B^	178^C^	2378^C^
ScIV	7.14 ^AB^ (7.90)	6.49^AB^	0.45^B^	16.04^AB^	557^D^	535^C^	151^B^	1243^D^	1169^A^	1469^B^	176^C^	2814^AB^
ScV	6.38^C^	6.61^AB^	0.51^B^	15.61^AB^	548^D^ (663)	510^C^ (625)	147^B^	1205^E^ (1435)	907^B^ (791)	1527^A^ (1412)	224^B^	2658^BC^ (2427)
ScVI	7.35^A^ (8.13)	6.79^A^	0.53^B^	16.85^A^	555^D^ (671)	509^C^ (625)	147^B^	1212^E^ (1442)	1219 ^A^ (1104)	1574^A^ (1459)	241^B^	3034^A^ (2804)

^a^Refer Table 5 for scenario description; 1 USD = 66.26 INR.

^b^Means followed by a similar uppercase letter(s) within a column in a given year are not significantly different at 0.05 level of probability using Tukey’s HSD test.

^c^Not applicable.

^*^Figures in parenthesis under grain yield represents the actual yield of maize. However, in cost of cultivation and net returns represents the actual values without SDI system subsidy (details are available in Supplementary Table 4).

On 2 years mean basis, highest system productivity (REY) of 16.0 Mg ha^−1^ was recorded with ScVI and the lowest (13.4 Mg ha^−1^) was with ScI (Table 1). Scenarios VI, II, IV and V recorded similar mean system productivity, which varied from 11.2 to 19.7% higher compared to CT (ScI). The CA^+^ practices in RW (ScV) and MW (ScVI) recorded on par system productivity similar to their respective CA-based practices with flood irrigation (ScIII and ScIV).

### Economic profitability

**Figure 1:**
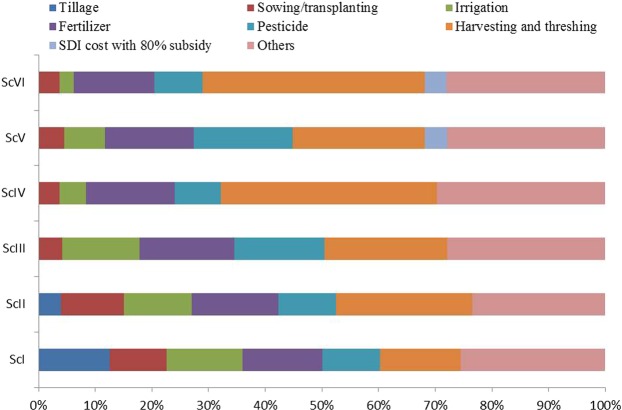
Cultivation cost (%) with subsidy on SDI system under different management scenarios in rice/maize based cropping systems (2-yr mean).

**Figure 2:**
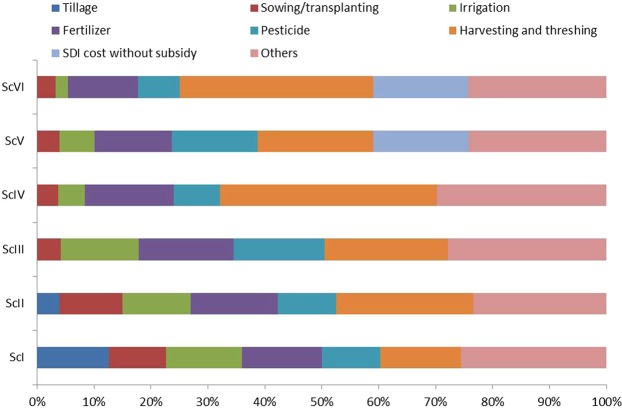
Cultivation cost (%) without subsidy on SDI system under different management scenarios in rice/maize based cropping systems (2-yr mean).

The higher cost of cultivation (tillage, sowing/transplanting, fertilizer, irrigation water, pesticides, harvesting and threshing etc.) was associated with CT based RW system (ScI) compared to CA and CA^+^- based systems (ScIII and ScV) during both the years (Table 1 and Supplementary Table 1). In ScI, 12.6% of total cost was incurred in tillage operation for seed bed preparation. Almost 15% of the total cost was associated with fertilizer use across the scenarios (Fig. 1). Harvesting and threshing contributed ~39% share in CA based maize systems and ~22% in CA based rice systems irrespective of irrigation management. The SDI system with subsidy (80%) and irrigation shared ~4% each to the total cost of cultivation (Fig. 1). However, the SDI system without subsidy incurred ~17% to the total cost of cultivation (Fig. 2) and the irrigation contributes merely 6 and 2% of total cost in CA^+^ ScV and ScVI, respectively. The cost of cultivation across two years of study ranged from USD 526–703 and 509–682 ha^−1^ in rice/maize and wheat crops, respectively (Table 1) with 80% subsidy on SDI system. However, without SDI subsidy, it ranged from USD 557–703 and 529–682 ha^−1^ in rice/maize and wheat, respectively (Supplementary Table 1). On a system basis, cultivation cost was 10.8% (2-yr mean) lower with CA-based management compared to CT-based system (ScI) irrespective of SDI system and mungbean integration. On 2 years mean basis, cultivation cost for SDI system with subsidy in R/M-W system (ScV and ScVI) was 12.9% lower (Table 1), however without subsidy the cultivation cost increased by 3.8% (Supplementary Table 1) compared to CT-based system (ScI). On 2 years mean basis, maize recorded the highest net returns (USD 1230 ha^−1^) and DSR recorded the lowest returns (USD 794 ha^−1^), irrespective of irrigation management. Maize recorded the highest net returns of USD 1254 and 1139 ha^−1^ (2-year mean) with and without SDI system subsidy. Lowest net returns were recorded with DSR flood irrigation (ScIII) during both the years. Significantly higher net returns from wheat were recorded with CA-based systems (ScIII and ScIV) in first year, while higher returns were recorded with SDI scenarios (CA^+^) in the second year. However, similar net returns were recorded from ScIII to VI in first year and ScII to ScVI in second year without subsidy on SDI system (Supplementary Table 1). The CA^+^ practices recorded 40 and 34% (2-yr mean) higher net returns in wheat compared to CT (USD 987.5 ha^−1^) system with and without SDI system subsidy. Higher net return in mungbean was recorded with ScII in both the years among compared to all the scenarios. Mean net returns in mungbean were 36% higher with SDI (ScV and ScVI) compared to flood irrigation system (ScI-IV).

In general, higher net returns (profitability) were recorded with maize-based systems compared to rice-based systems, irrespective of agronomic management practices. Highest mean profitability (USD 2791 ha^−1^) was recorded with the ScVI (maize-wheat-mungbean with CA+ with subsidy on SDI system). However, without subsidy on SDI system, it was higher with ScIV (Supplementary Table 1). The lowest profitability (USD 1874 ha^−1^) was recorded under ScI (Table 1). CA-based rice (mean of ScIII and ScV) and maize (mean of ScIV and ScVI) systems recorded 22 and 45% (2-yr mean) higher profitability compared to CT (USD 1874 USD ha^−1^), respectively irrespective of SDI system subsidy. The CA^+^ RW-mungbean (ScV) and MW-mungbean (ScVI) system recorded 12.5 and 5.4% (2-yr mean) higher profitability for SDI system with subsidy and recorded similar profitability without subsidy compared with their respective CA-based systems (ScIII and IV), respectively.

### Irrigation water use

Significantly higher (~1886 mm ha^−1^) amount of irrigation water (2-yr mean) was consumed by rice in the CT (ScI) compared to ScII (1620 mm ha^−1^) and ScIII (1447 mm ha^−1^) (Table 2). Maize consumed the lowest amount (85 mm) of irrigation water in ScVI. The CA^+^ practices saved 58 and 96% (2-yr mean) irrigation water in rice and maize crops compared with CT, respectively. The SDI system contributed 46 and 23% share in irrigation water saving with same management practices in rice and wheat, respectively. The CA practices in DSR (ScIII) saved 23% of irrigation water compared with ScI. Lesser amount of irrigation water (180–225 mm ha^−1^) was used with SDI system in wheat which resulted in saving of 53% of water over the CT (Table 2). Compared with CA, CA^+^ saved 11% of irrigation water in wheat. On 2 years mean, SDI system saved 55% (2-yr mean) of irrigation water compared with flood irrigation in mungbean.

**Table 2:** Irrigation water use and water productivity (WP_I_), and partial factor productivity on nitrogen (PFP_N_) under different scenarios during the year 2016–17 and 2017–18.

Scenarios^a^	Irrigation (mm ha^−1^)				WP_I_ (kg grain m^−3^)				PFP_N_ (kg grain kg^−1^ N applied)		
	Rice/Maize	Wheat	Mungbean	System	Rice/Maize	Wheat	Mungbean	System	Rice/Maize	Wheat	System
*2016–17*											
ScI	1893^Ab^	409^A^	-NA^c^-	2302^A^	0.40^C^	1.38^C^	-NA-	0.59^D^	42.91^C^	36.47^C^	41.23^C^
ScII	1562^B^	349^AB^	167^B^	2078^AB^	0.48^C^	1.64^BC^	0.30^A^	0.73^D^	49.27^B^	38.13^BC^	50.27^B^
ScIII	1420^B^	357^AB^	183^A^	1961^B^	0.42^C^	1.81^BC^	0.08^D^	0.67^D^	36.75^D^	42.33^B^	42.55^C^
ScIV	110^D^	337^B^	177^AB^	624^C^	7.18^B^	2.05^B^	0.09^D^	2.37^B^	40.69^C^	43.53^B^	44.98^C^
ScV	620^C^	188^C^	80^C^	888^C^	1.00^C^	3.64^A^	0.25^BC^	1.59^C^	47.77^B^	56.58^A^	56.48^A^
ScVI	86^D^	172^C^	80^C^	338^D^	9.54^A^	3.75^A^	0.27^B^	4.50^A^	54.36^A^	53.17^A^	58.23^A^
*2017–18*											
ScI	1878^A^	462^A^	-NA-	2340^A^	0.35^C^	1.28^B^	-NA-	0.57^E^	37.54^C^	39.20^B^	41.02^C^
ScII	1677^B^	452^A^	161^A^	2290^A^	0.41^C^	1.57^B^	0.46^B^	0.71^D^	45.67^AB^	40.40^B^	53.97^B^
ScIII	1474^C^	412^A^	160^A^	2046^B^	0.40^C^	1.63^B^	0.28^C^	0.73^D^	36.56^C^	43.87^B^	47.94^BC^
ScIV	110^E^	407^A^	161^A^	678^D^	7.19^B^	1.60^B^	0.28^C^	2.37^B^	40.80^B^	43.27^B^	49.35^B^
ScV	950^D^	226^B^	72^B^	1248^C^	0.67^C^	2.93^A^	0.70^A^	1.25^C^	49.08^A^	55.08^A^	62.44^A^
ScVI	84^E^	225^B^	72^B^	381^E^	9.68^A^	3.02^A^	0.73^A^	4.42^A^	52.50^A^	56.58^A^	64.81^A^

^a^Refer Table 5 for scenario description.

^b^Means followed by a similar uppercase letter(s) within a column are not significantly different at 0.05 level of probability using Tukey’s HSD test.

^c^Not applicable.

Note: Rainfall (mm) Rice: 433 (2016), 567 (2017); Maize: 433 (2016), 547 (2017); Wheat: 188 (2016–17), 170 (2017–18); Mungbean: 121 (2017), 76 (2018).

On system mean basis, ScI and ScII, consumed highest amount of irrigation water (mean of 2253 mm ha^−1^), while ScVI consumed the lowest (360 mm ha^−1^) amount of irrigation water compared to the other scenarios (Table 2). The CA^+^ practices saved 72 and 85% (2-yr mean) irrigation water in rice (ScV) and maize (ScVI) based systems compared to CT RW system (ScI), respectively. The CA^+^-based RW-mungbean (ScV) and MW-mungbean (ScVI) saved 45–47% of irrigation water compared to their respective CA based flood irrigation systems (ScIII and ScIV).

### Water productivity (WP)

The higher irrigation water productivity (WP_I_) was recorded with CA^+^ in both rice (ScV) and maize (ScVI) based systems compared to other scenarios during both the years (Table 2). On 2 years mean basis, highest WP_I_ in rice (9.61 kg grain m^−3^) was recorded with ScV (CA^+^) and lowest was with ScI (0.38 kg grain m^−3^). The CA^+^ practices in rice (ScV) and maize (ScVI) improved the mean WP_I_ by 123 and 246% compared with the ScI, respectively. Like rice and maize, the higher WP_I_ of wheat was observed with SDI compared to flood irrigation in both CA (ScII) and CT (ScI) in both the years (Table 2). The CA^+^ system improved the mean WP_I_ by 151% compared to CT in wheat (1.33 kg grain m^−3^). The corresponding increase with CA over CT was 29%. The WP_I_ of mungbean (2-yr mean) ranged from 0.18 to 0.50 kg grain m^−3^ across the scenarios. Highest (0.50 kg grain m^−3^) WP_I_ in mungbean was recorded in ScVI and lowest (0.18 kg grain m^−3^) in ScIII (Table 2).

On a system basis, the highest (4.46 kg grain m^−3^) WP_I_ was recorded with ScVI (CA^+^) and lowest (0.58 kg grain m^−3^) with ScI (Farmers’ practice) (Table 2). Maize-based Sc IV and ScVI recorded 145 and 669% higher mean WP_I_ compared to ScI, respectively. The corresponding increase in WP_I_ under ScII, ScIII and ScV ranged from 21–31%. The CA^+^ based RW-mungbean (ScV) and MW-mungbean (ScVI) recorded 103 and 88% (2-yr mean) higher WP_I_ compared to their respective CA based flood irrigation systems (ScIII and ScIV), respectively.

### Fertilizer-N use efficiency

The partial factor productivity of N (PFP_N_), an index of N use efficiency (NUE), was higher in the CA^+^ in both rice (ScV) and maize (ScVI) based systems compared to their respective CA and/or CT-based systems in both the years (Table 2). The highest (53.4 kg grain kg^−1^ N applied) PFP_N_ in maize was recorded with ScVI followed by rice (48.4 kg grain kg^−1^ N applied) in ScV (CA^+^) and lowest was in ScI (40.2 kg grain kg^−1^ N applied). The CA^+^ practices in rice (ScV) and maize (ScVI) improved the PFP_N_ by 20 and 33% (2-yr mean) compared to ScI in rice. Likewise, the PFP_N_ in wheat was significantly higher with the SDI compared to the flood irrigation in both partial CA (ScII) and CT (ScI) during both the years (Table 2). The CA^+^ system in wheat improved the PFP_N_ by 46% (2-yr mean) compared to the CT (37.8 kg grain kg^−1^ N applied).

On a system basis, the PFP_N_ was similar (~60.5 kg grain kg^−1^ N applied) (2-yr mean) for the CA^+^-based systems (ScV and ScVI) but significantly higher compared to ScI (41.12 kg grain kg^−1^ N applied) (Table 2). The CA^+^-based RW-mungbean (ScV) and MW-mungbean (ScVI) recorded 45 and 50% higher PFP_N_ compared to those of ScI and ~31% higher compared to their respective CA-based systems with flood irrigation (ScIII and ScIV), respectively.

### Energy use efficiency and productivity

Energy input, output, use efficiency (EUE) and productivity (EP) were affected by different management scenarios (Table 3). Higher energy (47.6 × 10^3^ MJ ha^−1^) was consumed in transplanted rice (ScI) compared with all other scenarios and the lowest (26.0 × 10^3^ MJ ha^−1^) energy input was recorded in CA^+^ rice (ScV). Energy input was similar for ScIV and ScVI in maize in both the years (Tale 8). In wheat, significantly lower energy input was recorded with the SDI (16.2 × 10^3^ MJ ha^−1^) compared with all other scenarios. Like rice, highest mean energy input (24.9 × 10^3^ MJ ha^−1^) in wheat was recoded in ScI followed by ScII, ScIII and ScIV. On a system basis, the CA^+^ practices saved 38 and 55% of energy input in rice- (ScV) and maize- (ScVI) based systems respectively, whereas the CA systems with flood irrigation saved 17 and 48% (2-yr mean) energy inputs compared to ScI, respectively.

**Table 3:** Effect of management scenarios on energy (input and output), energy use efficiency and productivity during 2016–17 and 2017–18.

Scenarios^a^	Energy input × 10^3^ MJ ha^−1^				Energy output × 10^3^ MJ ha^−1^				Energy use efficiency (MJ ha^−1^)				Energy productivity (kg MJ^−1^)			
	Rice/Maize	Wheat	Mung-bean	System	Rice/Maize	Wheat	Mung-bean	System	Rice/Maize	Wheat	Mung-bean	System	Rice/Maize	Wheat	Mung-bean	System
**2016–17**																
ScI	47.72^Ab^	24.16^A^	-NA^c^-	71.88^A^	224.6^A^	129.8^C^	-NA-	354.4^A^	4.71^E^	5.38^D^	-NA-	4.93^E^	0.16^D^	0.23^D^	0.00	0.18^D^
ScII	40.25^B^	18.79^B^	4.04^A^	66.44^B^	225.2^A^	132.6^BC^	7.40^A^	357.8^A^	5.63^D^	7.06^C^	1.83^A^	5.40^DE^	0.18^D^	0.30^C^	0.12^A^	0.21^DE^
ScIII	36.75^B^	19.12^B^	3.96^A^	58.13^C^	185.1^B^	153.0^A^	2.25^D^	338.2^A^	5.04^DE^	8.01^BC^	0.57^C^	5.82^D^	0.16^D^	0.33^C^	0.04^C^	0.21^E^
ScIV	15.14^D^	18.48^B^	3.88^A^	35.87^E^	1980^B^	150.9^A^	2.25^D^	348.9^A^	13.08^B^	8.19^B^	0.58^C^	9.73^B^	0.51^B^	0.36^C^	0.04^C^	0.40^B^
ScV	23.57^C^	16.08^C^	3.03^B^	42.54^D^	196.0^B^	154.1^A^	2.89^C^	350.0^A^	8.31^C^	9.59^A^	0.96^B^	8.23^C^	0.26^C^	0.42^A^	0.07^B^	0.31^C^
ScVI	13.13^D^	15.78^C^	3.03^B^	32.10^E^	199.8^B^	149.1^AB^	3.19^B^	348.9^A^	15.22^A^	9.45^A^	1.05^B^	10.87^A^	0.62^A^	0.40^AB^	0.07^B^	0.46^A^
**2017–18**																
ScI	47.55^A^	25.68^A^	-NA-	73.23^A^	213.o^BC^	142.8^A^	-NA-	355.8^B^	4.48^D^	5.56^C^	-NA-	4.86^E^	0.14^D^	0.23^C^	-NA-	0.18^E^
ScII	41.93^B^	21.26^B^	3.96^A^	67.15^B^	222.0^B^	146.6^A^	10.88^A^	379.5^B^	5.29^D^	6.98^B^	2.75^A^	5.65^D^	0.16^D^	0.29^B^	0.19^A^	0.24^D^
ScIII	37.61^C^	20.62^B^	3.87^A^	62.10^C^	192.6^C^	154.7^A^	6.62^B^	353.9^B^	5.14^D^	7.52^B^	1.71^C^	5.70^D^	0.16^D^	0.32^B^	0.12^C^	0.24^D^
ScIV	15.11^E^	20.50^B^	3.87^A^	39.49^E^	267.5^A^	154.7^A^	6.57^C^	428.8^A^	17.70^B^	7.56^B^	1.70^C^	10.86^B^	0.52^B^	0.32^B^	0.12^C^	0.41^B^
ScV	28.39^D^	16.58^C^	3.23^B^	48.21^D^	200.2^C^	156.8^A^	7.45^B^	364.4^B^	7.05^C^	9.45^A^	2.31^B^	7.56^C^	0.22^C^	0.40^A^	0.16^B^	0.32^C^
ScVI	13.10^E^	16.51^C^	3.23^B^	32.84 ^F^	274.45^A^	159.3^A^	7.74^B^	441.5^A^	20.96^A^	9.65^A^	2.40^B^	13.45^A^	0.62^A^	0.41^A^	0.16^B^	0.51^A^

^a^Refer Table 5 for scenario description.

^b^Means followed by a similar uppercase letter(s) within a column are not significantly different at 0.05 level of probability using Tukey’s HSD test.

^c^Not applicable.

In rice, significantly higher energy output was recorded under the ScI and ScII (219 × 10^3^ MJ ha^−1^) compared to all other scenarios in 2016–17 (Table 3). Whereas in 2017–18, ScIV and ScVI recorded significantly higher energy output compared with the other scenarios. The lowest energy output was recorded with rice in ScIII, whereas highest (235 × 10^3^ MJ ha^−1^) with maize in ScVI (Table 3). In wheat, the CA^+^ and CA with flood irrigation system recorded 13.6 and 9.2% higher mean energy output compared to Sc1, respectively. In mungbean, ScII recorded significantly higher energy input (4.0 × 10^3^ MJ ha^−1^) and output (9.14 × 10^3^ MJ ha^−1^) compared with other scenarios in both the years (Table 3). Similar energy output was observed with rice and maize based systems across the scenarios. However, it was 10% higher with maize-based scenario compared with ScI.

Both EUE and EP were significantly higher in maize-based systems compared to rice-based systems, irrespective of management practices during both the years of study (Table 3). Replacing flood irrigation with SDI in CA-based scenarios (ScIII and IV) significantly increased EUE and EP in all the crops. The EUE and EP recorded in ScV were 294 and 313% higher in rice and 67 and 60% higher with ScVI in maize, respectively compared to ScI in rice (4.6 MJ ha^−1^and 0.15 kg MJ^−1^). The CA-based wheat cultivation with flood irrigation increased EUE and EP by about 39%, and by 74–77% with SDI compared to ScI (5.47 MJ ha^−1^and 0.23 kg MJ^−1^). Significantly higher EUE and EP were recorded with ScII in mungbean during both the years of study (Table 3). In maize-based system, ScIV recorded 110 and 125% higher EUE and EP, and in CA^+^ (ScVI) EUP and EP were increased further to148 and 169% compared to ScI, respectively. Similarly, CA (ScIII) and CA^+^ (ScV) based management scenarios improved the mean EUE by 8 and 61% and EP by 25 and 75.0% in rice based systems, respectively compared to farmers’ practice (EUE 4.9 MJ ha^−1^and EP 0.18 kg MJ^−1^).

## Discussion

In NW India, rice-wheat system is mainly dependent on groundwater, extracting groundwater faster than it is replenished naturally by rainwater thus causing water tables to decline unremittingly^18^. Rodell *et al*.^19^ reported that unsustainable consumption of groundwater for agriculture crop production and other anthropogenic uses is the main cause of groundwater depletion in NW India. Furthermore, projected climate change and open access market will have serious impacts on groundwater resources and agriculture crop production in the future. Access to good quality irrigation water is key to reduce the impacts of stresses to achieve the food security in the region. In India, agriculture sector consumes ~70% of the total fresh water and it is affected by the rainfall pattern. However, with increasing population, urbanization and industrial growth, irrigated agriculture is being called on to ‘More Crop Per Drop’ to produce sustainably while protecting soil and water resources^20^. With the faster depleting ground water, water availability is bound to decline which may not only have negative impacts on future food security but may also lead to social unrest.

The rice yields of DSR in ScIII and ScV, were lower than that of the transplanted rice (ScI) in both the years (Table 1). Based on available datasets, Kumar and Ladha^21^ reported 10% lower yields in ZTDSR, to those of CT transplanted rice in India. Although, the mean yield with ZTDSR was about 14% lower (mean for ScIII and ScV, over 2-yr) than those of CT rice, however, ZTDSR has other advantages like savings in labor, time, water, energy, cost and yield advantage to succeeding crop in rotation and hence more profit^21^. Results from out study shows yields of DSR were higher under SDI by 0.4 Mg ha^−1^ (2-yr mean) compared to flood irrigation. Rice with CA recorded similar or higher yields under SDI systems with laterals spaced at 60 cm in other studies^15,17,22^. Compared with CA based DSR, maize recorded higher REY in both years (ScIV and ScVI). Results from our study showing lower production costs and higher profitability in CA based scenarios (rice/maize based systems) compared with CT are in consistent with the findings of other researchers^3,4,23^ in NW India. The higher profitability under CA^+^ (ScV and ScVI) owed mainly to combined effect of lesser amount of irrigation and fertilizer costs, and partly to higher crop productivity. In earlier studies Sivanappan^24^ and Sharda *et al*.^15^ in rice; Sidhu *et al*.^17^ in rice and wheat have also reported higher profitably under SDI compared with flood irrigation. The SDI system resulted in incremental trend in yield of maize compared with flood irrigation. Lamm and Trooien^25^ and Tarkalson *et al*.^26^ have recorded higher yield of maize under drip irrigation compared with flood irrigation at same rates of N fertilizer in semi-arid regions of North Plateau, Nebraska due to supply of water and N as per the crop demand. Considering increased efficiency of fertilizer N under SDI system, we applied 20% lower fertilizer N doses to all the crops in SDI compared with flood irrigation and yet harvesting higher yields. However, for precise information on fertilizer use efficiency under SDI vis-à-vis conventional flood irrigation systems warrants further investigation.

Retention of residues on soil surface under CA moderates soil temperature and conserves soil moisture by reducing evaporation losses which helps in increasing the availability of nutrients and water^4,17,27,28^. The SDI (ScV and ScVI) with CA helped in saving of 46, 23, 46 and 55% irrigation water in rice, maize, wheat and mungbean compared with CA with flood irrigation (ScIII and ScIV), respectively. Lower irrigation water use (72–85%) resulted in higher WP_I_ in all the crops compared with flood irrigation system (ScI) during both the years. Ramulu *et al*.^29^ and Sharda *et al*.^15^ recorded a 40–50% water savings and 90% higher WP with drip irrigation in DSR compared to farmers’ practice. The saving in irrigation water in SDI system was probably due to the reduced seepage and evaporation losses because of small amount (1–2 cm ha^−1^) of irrigation water applied in rice directly to the plant root zone at regular 2–5 days interval depending upon the tensiometer readings. In the SDI irrigation, water is applied as per the crop needs of cereals (rice, wheat and maize) which allows uniform distribution of soil moisture and nitrogen in the root zone, minimize the evaporative loss of water, and consequently saved irrigation water and increased WP as reported by other researchers^14,17,30–32^. On the other hand, flood irrigation (5–8 cm depth at each event) was prone to more percolation and evaporation losses because of higher amount of irrigation water applied than the soil water storage capacity^12,17^.

The higher N use efficiency in RW and MW systems under SDI using fertigation was achieved by effectively managing the placement and timing of fertilizer N in small amounts in more splits to match the crop demand and minimizing N losses through leaching, volatilization and denitrification. In our study, N was applied in 8 equal splits at 10 days intervals in SDI compared to that of 3 splits in rice and in 4 splits in wheat and maize in flood irrigation system. Due to efficient use and minimal losses, we used 20% lower fertilizer N which resulted in similar or higher productivity due to increase in N use efficiency compared to respective flood irrigation systems. Results from another study showed that fertigation saved ~50% of the recommended fertilizer N in rice over the flood irrigation system^22^. Also, Tarkalson *et al*.^26^ in maize and Sidhu *et al*.^17^ in RW system have reported significant increases in N use efficiency under SDI compared with flood irrigation system.

Higher EUE and EP in the CA^+^ rice/maize systems (ScV and VI) was due to lower input energy for tillage, fertilizer and irrigation and higher or similar energy output in terms of grain and straw yields compared to the other management scenarios. Under, CT rice-wheat and maize-wheat system, intensive tillage alone shares 33–40% of total energy (operational), which could be saved with ZT without affecting the crop yields. Similar energy savings were reported by many other researchers under CA based management systems in RW and MW systems^2–4^.

The SDI system proved to benefiting the farmers by increasing the water and N-use use efficiency through significant irrigation water and N-saving with similar or higher yields in cereal based systems^17,22^. But the SDI system requires higher initial investment than the flood irrigation system which may be a reason for low adoption without subsidy in the NW IGP where electricity is almost free for pumping groundwater. The water saving is affected by drip line depth and spacing^14^, emitter spacing and discharge, and soil texture. Although in this study we do not find any consequence of SDI but it was reported that tillage and pest (burrowing mammals, principally of the rodent family) may cause leakage/breakage that reduce SDI system uniformity^15^. Rodents are gnawing mammal acts on hardy materials such as plastic, wood etc. to wear down their continuously growing teeth^13^. Root intrusion and pinching of the drip line can affect wetting zone uniformity in the soil^30^. Clogging of emitters by fertigation and soil ingestion caused by back siphoning may occur under SDI system^25^. The adoption of SDI systems in cereal systems of IGP depends on the different Government policies like ‘More Crop Per Drop’, ‘Doubling Farm Income by 2022’, ‘Soil Health Mission’ and ‘Sustainable Agriculture’, and, to a large extent, on balance of potential advantages over potential disadvantages.

## Conclusions

In intensive cereal based systems of north-western Indo-Gangetic plains of South Asia, bundling precision agronomic innovations like conservation agriculture (CA), inclusion of short duration legumes coupled with subsurface drip irrigation (SDI) and fertigation provides science backed evidence to address multiple challenges of food, nutrition, water, energy, soil health & climate change. The CA^+^ (CA + SDI) based RW system increased the crop productivity and farm profitability by ~11 and 29%, respectively while saving of 72% of irrigation water compared to farmers’ practice (FP). The MW system with CA^+^ recorded ~20% higher productivity, 49% higher profitability and saved 85% of irrigation water compared to farmers’ practice/conventional RW system. Scheduling of fertilizer N through SDI (fertigation) in the CA^+^ based rice/maize-wheat systems saved 20% of N and increased N use efficiency by 47% compared with farmer’s practice of flood irrigation. The mungbean integration in cereal (RW/MW) systems contributed to 13.5 and 32.5% increase in productivity and profitability, respectively irrespective of cropping systems. Retaining crop residues as mulch to an alternative to burning and reduction in N losses with fertigation using SDI system in CA based cereal systems will help in reducing global warming potential in long run.

Considering the seriousness of speedily depleting groundwater resources, the provincial Governments in Punjab and Haryana as well as Government of India have initiated new policy program (Water is Life, Direct Benefit Transfer of Electricity etc.) for saving water in agriculture. However, there is a need for ‘Policy Driven Science’ for successful implementation of these policies and efficient use of investments. Therefore, results of our study on bundling of various complementing agronomic innovations including subsurface drip irrigation (SDI) systems for cereal crops would be of immense interest to farmers, policy planners and civil society for addressing the current and future challenges of farming.

## Methods

### Study site

A field study was conducted for 2 years (2016–17 and 2017–18) at ICAR-CSSRI (Indian Council of Agricultural Research- Central Soil Salinity Research Institute) – CIMMYT (International Maize & Wheat Improvement Centre) research platform ((29°70′N, 76°96′E)), Karnal, India. The experimental site represents the sub-tropical and semi-arid climate and characterized by three distinguished season i.e. *Kharif* (July-October), *Rabi* (November-March) and *Zaid* (April-June). The *Kharif* season (wet monsoon season) coincides with the South-West monsoon and received ~80% of total average annual rainfall (670 mm). The soil (0–15 cm layer) of the study site was loam in texture (34% sand, 46% silt and 20% clay) with a pH of 8.0 (1:2 soil: water). The soil was low in major available nutrients. The initial properties of the soil are given in Table 4.

**Table 4:** Initial soil (0–15 cm) properties of the experimental field in 2016–17.

Properties	Value	±SEm	Method Used
Sand (%)	34.0	0.77	Particle size analysis^33^
Silt (%)	46.1	0.76	
Clay (%)	19.9	0.50	
Textural class	Loam		USDA triangle
Bulk density (Mg m ^−3^)	1.54	0.03	Blake and Hartage^34^
Infiltration rate (cm hr^−1^)	0.14	0.03	Double ring infiltrometer method^35^
pH (1:2 soil: water)	8.00	0.02	Glass electrode pH meter^36^
EC (dS m^−1^)	0.30	0.02	Conductivity bridge^36^
Organic carbon (g kg^−1^)	7.6	0.11	Wet digestion method^37^
Available P (kg ha^−1^)	7.74	0.32	0.5 *M* NaHCO_3_ extractable^38^
Available N (kg ha^−1^)	250	9.5	Subbiah and Asija^39^
1 *M* Neutral NH_4_OAc- extractable K (kg ha^−1^)	167.0	4.73	Jackson^40^

### Experimental design and treatments

A long-term experiment included four cereal based scenarios (Sc) differing in cropping system, tillage and crop establishment, residue management, and other crop management practices was established in 2009–10 under CSISA (Cereal Systems Initiative for South Asia) project to address the issues of water, labor and energy in the IGP (Kumar et al., 2018). Four scenarios (Sc) included were: (i) conventional-till (CT) rice-CT wheat (ScI; farmers’ practice; CT); (ii) CT rice-Zero tillage (ZT) wheat-ZT mungbean with flood irrigation (ScII; partial CA); (iii) ZT rice-ZT wheat-ZT mungbean with flood irrigation (ScIII; CA); (iv) ZT maize-ZT wheat-ZT mungbean with flood irrigation (ScIV; CA). These scenarios were implemented in large-scale plots of 2000 m^2^ (100 m × 20 m) and replicated thrice in a randomized complete block design. A new set of two scenarios which included subsurface drip irrigation (SDI) was imposed in May 2016 by sub dividing the main plots of ScIII and ScIV into two sub plots {each sub plot size of 1000 m^2^ (50 m × 20 m)} and thus total scenarios were now six. The ScV was ZT rice-ZT wheat-ZT mungbean with SDI (ScV; CA^+^ RW) and the ScVI was ZT maize-ZT wheat-ZT mungbean with SDI (ScVI; CA^+^ MW). Details of all the scenarios including drivers of agricultural change along with different crop management practices are presented in Table 5.

**Table 5:** Drivers of change, crop rotation, tillage, crop establishment method, and residue and water management of different scenarios

Scenario	Drivers of Change	Crop Rotations	Tillage	Crop Establishment Method	Residue Management	Nutrient Management (NPK, kg/ha)	Water Management
I	Business as usual (Farmer’s Practice)	Rice-Wheat- Fallow	CT-CT	Rice: Transplanting Wheat: Broadcast	All residue removed	Rice: 175 + 58 + 0 Wheat: 150 + 58 + 0	Rice: Continuous flooding of 5-cm depth for 1 month, followed by irrigation applied at hair-line crack Wheat: Need based irrigation or at critical crop growth stages
II	Increase food production, income & nutrition through intensification and best management practices	Rice-Wheat-Mungbean	CT-ZT-ZT	Rice: Transplanting Wheat: Drill seeding Mungbean: Drill/relay	Full (100%) rice and anchored wheat residue retained on soil surface; full mungbean residue incorporated	Rice: 150 + 58 + 60 Wheat: 150 + 64 + 32 Mungbean: 0 + 0 + 0	Rice: Continuous flooding of 5-cm depth for first 15–20 days after transplanting followed by irrigation at −40 to −50 kPa matric potential at 15-cm depth till 1 week before flowering followed by irrigation at −15 to −20 kPa Wheat: Flood irrigation at −40 to −50 kPa matric potential
III	Deal with rising scarcity of labor, water, energy, malnutrition, degrading soil health and emerging climatic variability	Rice-Wheat-Mungbean	ZT-ZT-ZT	Rice: Drill seeding Wheat: Drill seeding Mungbean: Drill/relay	Full (100%) rice and mungbean; anchored wheat residue retained on soil surface	Rice: 160 + 64 + 62 Wheat: 150 + 64 + 32 Mungbean: 0 + 0 + 0	Rice: Kept soil wet for first 20 days followed by irrigation at −20 to −30 kPa matric potential Wheat: Flood irrigation at −40 to −0 kPa matric potential
IV	Sustainable intensification (SI) with futuristic cropping system to deal with same issues as in scenario 3	Maize-Wheat- Mungbean	ZT-ZT-ZT	Maize: Drill seeding Wheat: Drill seeding Mungbean: Drill/relay	Maize (65%) and full mungbean; anchored wheat residue retained on soil surface	Maize: 175 + 64 + 62 Wheat: 150 + 64 + 32 Mungbean: 0 + 0 + 0	Flood Irrigation at −50 kPa in maize and −40 to −0 kPa matric potential
V	SI of RW system with CA + to deal with same issues as in scenario 3	Rice-Wheat-Mungbean	ZT-ZT-ZT	Same as in scenario 3	Same as in scenario 3	Rice: 130 + 64 + 62 Wheat: 120 + 64 + 32 Mungbean: 0 + 0 + 0 N in rice- 8 splits & wheat- 4 splits through SD Fertigation	Subsurface drip irrigation (SDI) at −20 to −30 kPa in rice and −40 to −0 kPa matric potential in wheat
VI	SI of MW systems through CA + to deal same issues as in scenario 3	Maize-Wheat- Mungbean	ZT-ZT-ZT	Same as in scenario 4	Same as in scenario 4	Maize: 140 + 64 + 62 Wheat: 120 + 64 + 32 Mungbean: 0 + 0 + 0 N in maize- 3 splits & wheat- 4 splits through SD Fertigation	Subsurface drip irrigation (SDI) at −50 kPa in maize and −40 to −0 kPa matric potential in wheat

*Where*: CT- conventional tillage; ZT- zero tillage; CA- conservation agriculture; SI- sustainable intensification; SD- subsurface drip; SDI- subsurface drip irrigation; CA^+^- CA + SDI; N- nitrogen; P- phosphorus; K- potassium.

### Crop management (crop establishment, seed rate, sowing)

In ScI (farmers’ practice or business-as-usual) both rice and wheat crop were established with conventional practices. In CT rice plots, dry tillage comprised of two harrowings and two cultivators followed by wooden planking, and wet tillage (puddling) comprising of two harrowings and one planking operations. In CT wheat, preparatory tillage for seed bed preparation included 2 passes each of harrowing and cultivator followed by planking. Rice seedlings (25–30 days old) were transplanted manually in a random geometry (20 cm × 15 cm) in puddled fields and wheat was sown by manual broadcasting in tilled soil under ScI. In ScII, rice was transplanted in case of ScI after wet tillage operations comprising 3 passes of harrowing and one planking in standing mungbean crop after manual picking of mature pods. In other scenarios (ZT conditions), all the crops (direct seeded rice, wheat, mungbean) were planted with a row spacing of 22.5 cm using Happy Seeder with inclined plate seed metering mechanism. Maize was seeded by Happy Seeder at a seed to seed spacing of 20 cm and row spacing of 67.5 cm. Rice hybrid (Arize 6129) was seeded with a seed rate of 10 kg ha^−1^ in CT plots and 20 kg ha^−1^ in ZT plots. However, both CT and ZT wheat (HD 2967) were seeded at 120 and 100 kg seed rate ha^−1^, respectively. Both maize hybrid (DKC 9125) and mungbean (SML 668) was seeded with a seed rate of 20 kg ha^−1^. Prior to seeding, seeds of all the cereal crops were treated with fungicides, tabuconazole (Raxil 60 FS) and imidachloropid (Guicho 600 FS) at 1 ml kg^−1^ and 5 g kg^−1^ seed, respectively for controlling of incidence of diseases. Rice was seeded in the third and first week of June during 2016 and 2017, respectively under both the CT and ZT plots. However, maize crop was seeded in the third week of June every year before the onset of monsoon. The wheat was sown in the last week of October and short duration mungbean was grown between wheat harvest and rice sowing from the mid-April to mid-June every year.

### Crop residue management

In the farmers’ practice (ScI) all the residues from rice and wheat were removed from the ground level. In ScII, full (100%) residue of rice and anchored residue of wheat (20–22 cm above the ground level; 30%) were retained, while full residue of mungbean was incorporated during puddling operation for rice. In ScIII and ScV, full rice and mungbean, and anchored wheat residues were retained. However, in maize based systems (ScIV and ScVI) partial (65%) residue of maize, anchored wheat residue and full residue of mungbean were retained. Total amount of crop residue in different scenarios ranged from 22.31 to 26.17 Mg ha^−1^ in two years of study (Table 6). Scenario II received highest (26.17 Mg ha^−1^) amount of crops residue. CA and CA^+^-based RW-mungbean systems (ScIII and ScV) received almost equal amount (24 Mg ha^−1^) of crop residues. The CA and CA^+^- based MW-mungbean system (ScIV and ScVI) also received the similar (22 Mg ha^−1^) amount of crop residues. In ScII, mungbean residue (1.65 to 4.57 Mg ha^−1^) was incorporated during puddling operation (Table 6).

**Table 6:** Total residue load (Mg ha^−1^) under different scenarios over the years.

Scenarios^a^	Residue incorporated/retained (Mg ha^−1^)								
	2016–17				2017–18				Grand total
	Rice	Wheat	Mungbean	System	Rice	Wheat	Mungbean	System	
ScI	-NA^b^-	-NA-	-NA-	-NA-	-NA-	-NA-	-NA-	-NA-	-NA-
ScII	7.93	0.99	1.65	10.57	8.25	2.78	4.57	15.6	26.2
ScIII	6.71	1.22	1.80	9.73	7.25	2.77	4.25	14.3	24.0
ScIV	5.80	1.12	2.20	9.12	6.69	2.49	4.15	13.3	22.5
ScV	7.12	1.11	2.23	10.46	7.24	2.48	4.27	14.0	24.5
ScVI	5.39	1.13	2.30	8.82	7.01	2.55	3.93	13.5	22.3

^a^Refer Table 5 for scenario description.

^b^Not applicable.

### Fertilizer management

The NPK doses (nitrogen, phosphorus and potash) were applied as per the recommendation of CCS Haryana Agricultural University, Hisar, India for both rice and wheat crops (150:60:60 kg ha^−1^ of N: P_2_O_5_: K_2_O), but farmers in this region apply excess N (175 kg ha^−1^) but no K (Table 5). Nutrient doses for N, P and K for all the scenarios are given in Table 5. The N was supplied mainly through urea (46% N) and partially as basal through DAP (di-ammonium phosphate) (18:46:00) and NPK (12:32:16) complex fertilizer in different scenarios. However, P and K were applied as basal through DAP and MoP (muriate of potash- 60% K_2_O), respectively in all the scenarios. In ScI, 125 kg DAP as basal and 330 kg urea (46% N) ha^−1^ as top-dressed in three equal splits was applied at early establishment (7–10 DAT; days after transplanting), active tillering (22–25 DAT), and panicle initiation stage (45–50 DAT) in rice. In CT wheat, 125 kg DAP was applied as basal and 150 kg ha^−1^ urea at crown root initiation (21–23 DAS-days after sowing) and 125 kg ha^−1^ urea at maximum tillering stage (45 DAS) was applied manually. In ScII, 125 kg DAP and 100 kg MOP ha^−1^ was applied as basal and 280 kg urea ha^−1^ was top-dressed in three equal splits as in ScI in rice. To wheat, 200 kg ha^−1^ of NPK (12:32:16) was applied as basal and urea was top-dressed as in ScI. In ScIII, 200 kg NPK ha^−1^ was drilled as basal and 300 kg urea ha^−1^ was applied in three splits of 50 kg ha^−1^ at early crop establishment (14–16 days after sowing, DAS), 125 kg ha^−1^ each at active tillering (23–25 DAS) and panicle initiation (50–55 DAS) stage in direct seeded rice (DSR). However, in ScIII and V, wheat crop was fertilized as in ScII. In ScIV, 200 kg NPK ha^−1^ was drilled at sowing and 325 kg ha^−1^ urea was top-dressed in three splits of 125, 125 and 75 kg ha^−1^ at 20, 45 and 60 DAS, respectively. In subsurface drip irrigated scenario (ScV and ScVI), 80% of the total N as urea (minus N added through DAP and NPK complex) was applied through fertigation at 10 days intervals in eight equal splits staring at 15 DAS in rice, and in maize (ScVI) it was applied in four equal splits at 20, 30, 45 and 60 DAS. Subsurface drip irrigated wheat (ScV and ScVI) received the 80% of the total N as urea in four equal splits through fertigation at 25, 45, 65 and 85 DAS. In mungbean no fertilizer was applied in any of the scenarios.

To determine the efficiency of applied N, partial factor productivity (PFP_N_) was calculated using Eq. 1 and expressed in kg grain per kg of N applied.

1 $${{\rm{PFP}}}_{{\rm{N}}}={\rm{Y}}\,({\rm{grain}}\,{\rm{yield}}\,{\rm{of}}\,{\rm{crop}}/{\rm{cropping}}\,{\rm{system}},\,{\rm{kg}}\,{{\rm{ha}}}^{-1})/{\rm{F}}\,({\rm{N}}\,{\rm{applied}},\,{\rm{kg}}\,{{\rm{ha}}}^{-1})$$

### Installation of subsurface drip irrigation system

The subsurface drip irrigation (SDI) system consisted of main line, sub-mains and laterals with an inside diameter of 90, 63 and 16 mm, respectively. The polyethylene laterals were laid parallel to the planting rows. After the Venturi injector, the water meter (Dasmesh Co., India) was fitted at the starting point of main line to measure the volume of water applied to each plot. The operating pressure for drip irrigation was maintained between 1.5 to 2.0 kg cm^−2^. The emitters were in-line type and having a capacity of 2.0 L h^−1^ at a pressure of 135 kPa and spaced at 30 cm. The mains and sub-mains were laid out at 100 cm, while lateral at the depth of 20 cm using tractor operated drip laying machine. The drip laying machine was developed by BISA, Ludhiana, India^17^. The lateral depths for SDI system in RW and MW system was based on the results from a study conducted at BISA, Ludhiana. The laterals were spaced at 67.5 cm in both rice and maize based systems. Each dripline served three rows (67.5 cm) of rice, wheat and mungbean and one row of maize crop. Drip system consisted of hydro cyclone filter and fitted at the source of irrigation. For fertigation, a Venturi injector was used and the suction was created due to the pressure differences between upstream and downstream.

### Water management

In the rice, maize and wheat crops, irrigation was applied on the basis of tensiometer (IRROMETER, River-side, California) readings (soil moisture potential; SMP) installed at 15 cm soil depth between the laterals and/or crop rows. The values of SMP used for all the crops under different management scenarios are presented in Table 5. In CT rice, continuous flooding was maintained for first three weeks for proper establishment of transplanted seedlings. In DSR, irrigation was applied frequently from sowing until the 3–4 leaf stage to ensure proper germination and crop establishment and subsequent irrigations were scheduled according to threshold value of SMP. The PVC pipeline was used to irrigate the crops under flood irrigation system and the water was delivered to each plot to avoid conveyance losses. The PVC pipeline was connected to a tube well with a water meter fitted in the outlet pipe. Each outlet (plot inlet) at plot had a butterfly valve to control irrigation water. In SDI, the water meter is fitted after the venturi injector in between the horizontal pipe just above the ground to make ease in taking readings. All the three replications of a particular scenario received similar amount of irrigation water at the same time. The volume of water applied in flood and SDI plots for each crops in each irrigation was measured using water meter (Landforce Dasmesh Mechanical Works, Punjab, India) fitted on the delivery pipe. The amount of total water consumed by the particular crop was calculated by summing the irrigation water and rainfall. Rainfall was measured using a rain gauge installed in the meteorological laboratory near to the site. The amount of irrigation water that was applied was quantified (in mm ha^−1^) by using Eqs. 2 and 3, while, irrigation water productivity (WP_I_) was calculated using Eq. 4 as shown below:

2 $$\begin{array}{c}{\rm{Volume}}\,{\rm{of}}\,{\rm{irrigation}}\,{\rm{water}}\,({\rm{kilolitre}}\,{{\rm{ha}}}^{-1})=\{({\rm{Final}}\,{\rm{water}}\,{\rm{meter}}\,{\rm{reading}}\\ \,\,-\,{\rm{Initial}}\,{\rm{water}}\,{\rm{meter}}\,{\rm{reading}})/{\rm{Plot}}\,{\rm{area}}\,{\rm{in}}\,{{\rm{m}}}^{2}\}\ast 10000\end{array}$$

3 $${\rm{Irrigation}}\,{\rm{water}}\,({\rm{mm}}\,{{\rm{ha}}}^{-1})={\rm{Volume}}\,{\rm{of}}\,{\rm{irrigation}}\,{\rm{water}}\,({\rm{kilolitre}}\,{{\rm{ha}}}^{-1})/10$$

4 $${{\rm{WP}}}_{{\rm{I}}}({\rm{kg}}\,{\rm{grain}}\,{{\rm{m}}}^{-3})={\rm{Grain}}\,{\rm{yield}}\,({\rm{kg}}\,{{\rm{ha}}}^{-1})/{\rm{Irrigation}}\,{\rm{water}}\,{\rm{used}}\,({{\rm{m}}}^{3}{{\rm{ha}}}^{-1})$$

*Where*, 1 ha-mm irrigation depth = 10 kilolitres = 10 m^3^; 1 m^3^ = 1000 litre.

### Energy analysis

All the crop inputs including labor, machinery, seed, diesel, fertilizer, irrigation pesticides etc. were used to estimate the total energy input using energy equivalents (MJ unit^−1^) values (Table 7) in each crop and cropping system under different scenarios. To estimate the total energy outputs from all the cropping systems under different scenario, the energy equivalents (MJ unit^−1^) values (Table 7) of each output (grain and straw) were used. Energy use efficiency (EUE) (MJ ha^−1^) of crops and cropping system from the different scenarios was calculated as the ratio between total energy outputs and total energy inputs. Based on the energy equivalents of the inputs and grain outputs (Table 7), energy productivity (EP) were calculated using the Eqs. 5 and 6.

5 $${\rm{Energy}}\,{\rm{use}}\,{\rm{efficiency}}={\rm{Total}}\,{\rm{energy}}\,{\rm{Output}}\,({\rm{MJ}}\,{{\rm{ha}}}^{-1})/{\rm{Total}}\,{\rm{energy}}\,{\rm{Input}}\,({\rm{MJ}}\,{{\rm{ha}}}^{-1})$$

6 $${\rm{Energy}}\,{\rm{productivity}}\,({\rm{kg}}\,{{\rm{MJ}}}^{-1})={\rm{Grain}}\,{\rm{output}}\,({\rm{kg}}\,{{\rm{ha}}}^{-{\rm{1}}})/{\rm{Total}}\,{\rm{energy}}\,{\rm{input}}\,({\rm{MJ}}\,{{\rm{ha}}}^{-1})$$

**Table 7:** Energy equivalents (MJ unit^−1^) used for energy input and output calculations.

Particulars	Units	Unit energy equivalent (MJ Unit^−1^)	References
*Input*			
Human labour	Man-hour	1.96	Gathala *et al*.^41^
Diesel	Litre	56.31	Gathala *et al*.^41^
Nitrogen (N)	kg	66.14	Gathala *et al*.^41^
Phosphorus (P_2_O_5_)	kg	12.44	Gathala *et al*.^41^
Potassium (K_2_O)	kg	11.15	Gathala *et al*.^41^
Herbicides, insecticides and pesticides	kg	120.00	Gathala *et al*.^41^
Irrigation water	ha-cm	143.56	Gathala *et al*.^41^
Zinc sulphate (ZnSO_4_)	kg	8.40	Argiro *et al*.^42^
Iron sulphate (FeSO_4_)	kg	110.00	Argiro *et al*.^42^
Rice, maize, wheat and mungbean	kg	14.70	Ozkan *et al*.^43^
*Output*			
Rice, maize, wheat and mungbean grain	kg	14.70	Ozkan *et al*.^43^
Rice, maize, wheat and mungbean	kg	12.50	Ozkan *et al*.^43^

### Economic analysis

The variable costs was calculated by taking in to account for the different crop operations (e.g. nursery raising for rice, tillage for seed bed preparation in wheat, seeding of DSR, wheat, maize and mungbean, harvesting and threshing of all crops), and inputs (seed, fertilizers, irrigation, herbicides, pesticides and labor for different operations) for raising the crops (Table 8). The labors required for different crop operations like rice transplanting, weeding, use of agrochemicals, water and fertilizer application, harvesting, threshing etc., were recorded by considering 8 hours a day equivalent to one person-days ha^−1^. Similarly, the mechanical operations (e.g. tillage, seeding, harvesting and threshing) performed by tractor and combine harvester was expressed as hr ha^−1^. The prices of all the inputs used for crop production were recorded. The irrigation water was charged at INR 0.30 per kWh of electricity fixed by local government of Haryana. The irrigation costs included the labor charges used for irrigation application. Cost of drip irrigation system was calculated based on both with and without subsidy. Govt of India offered the 80% subsidy on installation of SDI system on the actual cost basis (INR 250 × 10^3^ ha^−1^). The fixed cost of the drip system was computed by considering the life span of different parts. For main, sub-main, venturi, and pump, 20 years of life span was considered. However, the life span of polyethylene laterals was considered 15 yrs. Depreciation cost for the SDI system was considered at 10% each year. The gross returns (GR) were calculated by summing the income from the sale of grain and straw of rice, maize, wheat and mungbean. The market price of grain crops was based on the minimum support price (MSP) that was fixed by the Govt. of India every year and prevailing local market rates were used for crops straw (Table 8). The net return from each crop was calculated after subtracting variable costs/cultivation costs from the calculated GR. The system net returns were estimated by summing the net returns of all crops harvested within a calendar year. Indian rupee (INR) was converted to US$ (USD) based on a conversion rate of INR for 1 US$ for the respective years.

**Table 8:** Cost of key inputs and outputs used for economic analysis during the different years.

Item/Commodity	Cost (INR; Indian Rupee)	
	2016–17	2017–18
Rice grain (kg^−1^)	14.7	15.1
Rice straw (kg^−1^)	-NA-	-NA-
Rice seed (kg^−1^)	300	300
Maize grain (kg^−1^)	13.65	13.65
Maize straw (kg^−1^)	1.80	1.80
Maize seed (kg^−1^)	300	300
Wheat grain (kg^−1^)	15.10	17.35
Wheat straw (kg^−1^)	5.00	5.00
Wheat seed (kg^−1^) HD2967	32.0	32.0
Mungbean grain (kg^−1^)	48.5	48.5
Mungbean straw (kg^−1^)	-NA-	-NA-
Mungbean seed (kg^−1^) SML 668	50.0	50.0
Urea (kg^−1^)	5.6	5.6
Di-ammonium-phosphate (DAP) (kg^−1^)	-NA-	-NA-
Muriate of potash (MOP) (kg^−1^)	-NA-	-NA-
NPK complex (kg^−1^)	21.7	21.7
Zinc sulphate (ZnSO_4_) (kg^−1^)	-NA-	-NA-
Diesel (l^−1^)	55.2	60.0
Wages rate (person^−1^ day^−1^)	350	360
Subsurface drip cost with 80% subsidy and 15-years of life (ha^−1^ per principle crops)	1530	1530
Subsurface drip cost without subsidy and 15-years of life (ha^−1^ per principle crops)	7650	7650
USD conversion rate	66.26	66.26

^a^Refer Table 5 for scenario description.

^b^Not applicable,

### Crop yields and system productivity

At maturity, crops were harvested manually for grain and straw yields according to residue management protocols (Table 5). The samples (randomly) from rice and wheat were taken from a total area of 50 m^2^ from each plot from two locations of 25 m^2^ each. Maize samples were taken from a total area of 60 m^2^ from each plot from two locations of 30 m^2^ each. Grain yield was expressed as Mg ha^−1^ at 14%, 12%, and 14% grain moisture content for rice, wheat, and maize, respectively. Mungbean yields were recorded by manually picking the pods of entire plot. Grain yields of maize, rice, wheat and mungbean were converted to the rice equivalent yield (REY) by using Eq. 7.

7 $$\begin{array}{c}{\rm{Rice}}\,{\rm{equivalent}}\,{\rm{yield}}\,({\rm{Mg}}\,{{\rm{ha}}}^{-1})=[{\rm{maize}}/{\rm{wheat}}/{\rm{mungbean}}\,{\rm{yield}}\,({\rm{Mg}}\,{{\rm{ha}}}^{-1})\\ \times {\rm{MSP}}\,{\rm{of}}\,{\rm{respect}}{\rm{ive}}\,{\rm{crop}}\,({\rm{INR}}\,{{\rm{Mg}}}^{-1})]/\mathrm{MSP}\,{\rm{of}}\,{\rm{rice}}\,({\rm{INR}}\,{{\rm{Mg}}}^{-1})]\end{array}$$

*Where*, MSP- Minimum support price of Govt. of India; INR- Indian Rupee

### Data analysis

The data for different parameters were analyzed by ANOVA (analysis of variance) using SAS 9.1 software (SAS Institute, Cary, NC). Fisher’s least significant difference (LSD) test was used for comparing differences between two scenario means at the 5% probability level.

## Supplementary information

supplementary tables
