# Supplementary material for: Re-designing irrigated intensive cereal systems through bundling precision agronomic innovations for transitioning towards agricultural sustainability in North-West India
Source: Sci Rep. 2019 Nov 29;9:17929. doi: 10.1038/s41598-019-54086-1 (PMC6884493; doi:10.1038/s41598-019-54086-1)
Supplement: Supplementary file 1 — supplementary tables [file 41598_2019_54086_MOESM1_ESM.docx]

**Re-designing irrigated intensive cereal systems through bundling precision agronomic innovations for transitioning towards agricultural sustainability in North-West India**

H. S. Jat^1,2^, P. C. Sharma^1*^, A. Datta^1^, M. Choudhary^1^, S. K. Kakraliya^1^, Yadvinder-Singh^2,3^, Harminder S. Sidhu^3^, B. Gerard^4^ and M. L. Jat^2*^

^1^ICAR-Central Soil Salinity Research Institute (CSSRI), Karnal, Haryana, India

^2^International Maize and Wheat Improvement Center (CIMMYT), New Delhi, India

^3^Borlaug Institute for South Asia (BISA)-CIMMYT, Ludhiana, India

^4^International Maize and Wheat Improvement Center (CIMMYT), El-Batan, Texcoco, Mexico

*Corresponding Authors:

Dr. PC Sharma, Director, ICAR-CSSRI, Karnal

**Tel:** +91 (184) 2290501; Mob**:** +91 9416296240

Email: [pcsharma.knl@gmail.com](mailto:pcsharma.knl@gmail.com)

Dr. ML Jat, Principal Scientist/Systems Agronomist, CIMMYT-India

CG Block, NASC Complex, Pusa, New Delhi- 110 012, India

**Tel:** +91 (11) 55441938 Mob**:** +91 9999108787

Email: [m.jat@cgiar.org](mailto:m.jat@cgiar.org)

**Supplementary table 1**

Effect of management practices portfolios on cost of cultivation without subsidy on SDI system and net returns during year 2016-17 and 2017-18

| Scenarios^a^ | Grain yield (Mg ha^-1^) | | | | Cost of cultivation (USD ha^-1^) | | | | Net return (USD ha^-1^) | | | |
| --- | --- | --- | --- | --- | --- | --- | --- | --- | --- | --- | --- | --- |
|  | Rice equivalent | Wheat | Mungbean | System | Rice/Maize | Wheat | Mungbean | System | Rice/Maize | Wheat | Mungbean | System |
| *2016-17* | | | | | | | | | | | | |
| ScI | 7.51^Ab^ | 5.47^C^ | -NA^c^ - | 13.40^B^ | 687^A^ | 672^A^ | -NA- | 1359^B^ | 979^B^ | 828^C^ | -NA | 1807^C^ |
| ScII | 7.39^B^ | 5.72^BC^ | 0.50^A^ | 15.08^A^ | 655^AB^ | 531^C^ | 150^A^ | 1335^B^ | 984^B^ | 1021^BC^ | 219^A^ | 2225^B^ |
| ScIII | 5.88^C^ | 6.35^AB^ | 0.15^B^ | 13.19^B^ | 600^C^ | 529^C^ | 117^B^ | 1247^C^ | 705^C^ | 1225^AB^ | -5^B^ | 1925^C^ |
| ScIV | 7.12^AB^ (7.66) | 6.53^AB^ | 0.15^B^ | 14.62^A^ | 568^C^ | 527^C^ | 117^B^ | 1212^D^ | 1243^A^ | 1243^A^ | -5 ^B^ | 2482^A^ |
| ScV | 6.21^C^ | 6.79^A^ | 0.20^B^ | 14.12^AB^ | 642^B^ | 640^B^ | 114^B^ | 1395^A^ | 736^C^ | 1186^AB^ | 30^B^ | 1953^C^ |
| ScVI | 7.61^A^ (8.20) | 6.38^AB^ | 0.22^B^ | 15.14^A^ | 674^AB^ | 639^B^ | 114^B^ | 1426^A^ | 1173^A^ | 1098^AB^ | 45^B^ | 2317^AB^ |
| *2017-18* | | | | | | | | | | | | |
| ScI | 6.57^C^ | 5.88^C^ | -NA- | 13.33^C^ | 703^A^ | 682^A^ | -NA- | 1385^B^ | 795^C^ | 1147^B^ | -NA- | 1941^C^ |
| ScII | 6.85^BC^ | 6.06^BC^ | 0.74^A^ | 16.19^AB^ | 656^B^ | 536^C^ | 227^A^ | 1418^A^ | 905^BC^ | 1346^A^ | 315^A^ | 2567^AB^ |
| ScIII | 5.85^C^ | 6.58^AB^ | 0.45^B^ | 14.86^BC^ | 623^C^ | 532^C^ | 151^B^ | 1306^C^ | 710^C^ | 1490^A^ | 178^C^ | 2378^B^ |
| ScIV | 7.14 ^AB^ (7.90) | 6.49^AB^ | 0.45^B^ | 16.04^AB^ | 557^D^ | 535^C^ | 151^B^ | 1243^D^ | 1169^A^ | 1469^A^ | 176^C^ | 2814^A^ |
| ScV | 6.38^C^ | 6.61^AB^ | 0.51^B^ | 15.61^AB^ | 663^B^ | 625^B^ | 147^B^ | 1435^A^ | 791^C^ | 1412^A^ | 224^B^ | 2427^B^ |
| ScVI | 7.35^A^ (8.13) | 6.79^A^ | 0.53^B^ | 16.85^A^ | 671^B^ | 625^B^ | 147^B^ | 1442^A^ | 1104^AB^ | 1459^A^ | 241^B^ | 2804^A^ |

^a^Refer table 2 for scenario description; 1 USD=66.26 INR

^b^Means followed by a similar uppercase letter(s) within a column in a given year are not significantly different at 0.05 level of probability using Tukey’s HSD test

^c^Not applicable

^*^Figures in parenthesis are actual yield of maize

**Supplementary table 2**

Analysis of variance (ANOVA) showing degree of freedom and significance of the effects on grain yield, net returns, irrigation water, water productivity and energy indices of rice, maize, wheat, and rice-wheat and maize-wheat systems during 2016-17 and 2017-18.

**Grain yields**

Analysis of variance (ANOVA) for grain yield of rice/maize under different scenarios during year 2016

| **Source** | **DF** | **Type III SS** | **Mean Square** | **F Value** | **Pr > F** | **Significant** |
| --- | --- | --- | --- | --- | --- | --- |
| Replication | 2 | 0.5721 | 0.2860 | 1.3279 | 0.3080 | NS |
| Treatment | 5 | 7.9458 | 1.5892 | 7.3779 | 0.0039 | ** |
| Error | 10 | 2.1540 | 0.2154 | . | . | - |
| Corrected Total | 17 | 10.6718 | . | . | . | - |
| ** - Significant at 1%, * - Significant  at 5%, NS - Non Significant | | | | | | |

Analysis of variance (ANOVA) for grain yield of rice/maize under different scenarios during year 2017

| **Source** | **DF** | **Type III SS** | **Mean Square** | **F Value** | **Pr > F** | **Significant** |
| --- | --- | --- | --- | --- | --- | --- |
| Replication | 2 | 0.0367 | 0.0183 | 0.0515 | 0.9500 | NS |
| Treatment | 5 | 5.2164 | 1.0433 | 2.9314 | 0.0695 | NS |
| Error | 10 | 3.5589 | 0.3559 | . | . | - |
| Corrected Total | 17 | 8.8120 | . | . | . | - |
| ** - Significant at 1%, * - Significant  at 5%, NS - Non Significant | | | | | | |

Analysis of variance (ANOVA) for grain yield of wheat under different scenarios during year 2016-17

| **Source** | **DF** | **Type III SS** | **Mean Square** | **F Value** | **Pr > F** | **Significant** |
| --- | --- | --- | --- | --- | --- | --- |
| Replication | 2 | 0.2988 | 0.1494 | 0.6662 | 0.5351 | NS |
| Treatment | 5 | 3.8365 | 0.7673 | 3.4216 | 0.0463 | * |
| Error | 10 | 2.2426 | 0.2243 | . | . | - |
| Corrected Total | 17 | 6.3779 | . | . | . | - |
| ** - Significant at 1%, * - Significant  at 5%, NS - Non Significant | | | | | | |

Analysis of variance (ANOVA) for grain yield of wheat under different scenarios during year 2017-18

| **Source** | **DF** | **Type III SS** | **Mean Square** | **F Value** | **Pr > F** | **Significant** |
| --- | --- | --- | --- | --- | --- | --- |
| Replication | 2 | 0.3141 | 0.1570 | 1.4100 | 0.2888 | NS |
| Treatment | 5 | 1.8858 | 0.3772 | 3.3866 | 0.0476 | * |
| Error | 10 | 1.1137 | 0.1114 | . | . | - |
| Corrected Total | 17 | 3.3136 | . | . | . | - |
| ** - Significant at 1%, * - Significant  at 5%, NS - Non Significant | | | | | | |

Analysis of variance (ANOVA) for grain yield of systems under different scenarios during year 2016-17

| **Source** | **DF** | **Type III SS** | **Mean Square** | **F Value** | **Pr > F** | **Significant** |
| --- | --- | --- | --- | --- | --- | --- |
| Replication | 2 | 0.0435 | 0.0218 | 0.0542 | 0.9475 | NS |
| Treatment | 5 | 10.4650 | 2.0930 | 5.2161 | 0.0130 | * |
| Error | 10 | 4.0126 | 0.4013 | . | . | - |
| Corrected Total | 17 | 14.5212 | . | . | . | - |
| ** - Significant at 1%, * - Significant  at 5%, NS - Non Significant | | | | | | |

Analysis of variance (ANOVA) for grain yield of systems under different scenarios during year 2017-18

| **Source** | **DF** | **Type III SS** | **Mean Square** | **F Value** | **Pr > F** | **Significant** |
| --- | --- | --- | --- | --- | --- | --- |
| Replication | 2 | 0.5596 | 0.2798 | 0.3670 | 0.7018 | NS |
| Treatment | 5 | 23.2144 | 4.6429 | 6.0890 | 0.0077 | ** |
| Error | 10 | 7.6251 | 0.7625 | . | . | - |
| Corrected Total | 17 | 31.3991 | . | . | . | - |
| ** - Significant at 1%, * - Significant  at 5%, NS - Non Significant | | | | | | |

**Net returns**

Analysis of variance (ANOVA) for net returns of rice/maize under different scenarios during year 2016

| **Source** | **DF** | **Type III SS** | **Mean Square** | **F Value** | **Pr > F** | **Significant** |
| --- | --- | --- | --- | --- | --- | --- |
| Replication | 2 | 8071.3889 | 4035.6945 | 0.5450 | 0.5961 | NS |
| Treatment | 5 | 755802.1892 | 151160.4378 | 20.4137 | 0.0001 | ** |
| Error | 10 | 74048.5541 | 7404.8554 | . | . | - |
| Corrected Total | 17 | 837922.1322 | . | . | . | - |
| ** - Significant at 1%, * - Significant  at 5%, NS - Non Significant | | | | | | |

Analysis of variance (ANOVA) for net returns of rice/maize under different scenarios during year 2017

| **Source** | **DF** | **Type III SS** | **Mean Square** | **F Value** | **Pr > F** | **Significant** |
| --- | --- | --- | --- | --- | --- | --- |
| Replication | 2 | 8445.9903 | 4222.9952 | 0.2472 | 0.7856 | NS |
| Treatment | 5 | 618436.6141 | 123687.3228 | 7.2402 | 0.0041 | ** |
| Error | 10 | 170833.7789 | 17083.3779 | . | . | - |
| Corrected Total | 17 | 797716.3833 | . | . | . | - |
| ** - Significant at 1%, * - Significant  at 5%, NS - Non Significant | | | | | | |

Analysis of variance (ANOVA) for net returns of wheat under different scenarios during year 2016-17

| **Source** | **DF** | **Type III SS** | **Mean Square** | **F Value** | **Pr > F** | **Significant** |
| --- | --- | --- | --- | --- | --- | --- |
| Replication | 2 | 13410.7256 | 6705.3628 | 0.4809 | 0.6318 | NS |
| Treatment | 5 | 482538.8560 | 96507.7712 | 6.9212 | 0.0049 | ** |
| Error | 10 | 139438.8836 | 13943.8884 | . | . | - |
| Corrected Total | 17 | 635388.4653 | . | . | . | - |
| ** - Significant at 1%, * - Significant  at 5%, NS - Non Significant | | | | | | |

Analysis of variance (ANOVA) for net returns of wheat under different scenarios during year 2017-18

| **Source** | **DF** | **Type III SS** | **Mean Square** | **F Value** | **Pr > F** | **Significant** |
| --- | --- | --- | --- | --- | --- | --- |
| Replication | 2 | 4835.2676 | 2417.6338 | 0.2726 | 0.7669 | NS |
| Treatment | 5 | 367609.1744 | 73521.8349 | 8.2890 | 0.0025 | ** |
| Error | 10 | 88697.6060 | 8869.7606 | . | . | - |
| Corrected Total | 17 | 461142.0480 | . | . | . | - |
| ** - Significant at 1%, * - Significant  at 5%, NS - Non Significant | | | | | | |

Analysis of variance (ANOVA) for net returns of systems under different scenarios during year 2016-17

| **Source** | **DF** | **Type III SS** | **Mean Square** | **F Value** | **Pr > F** | **Significant** |
| --- | --- | --- | --- | --- | --- | --- |
| Replication | 2 | 3527.4203 | 1763.7101 | 0.0950 | 0.9101 | NS |
| Treatment | 5 | 1293581.2707 | 258716.2541 | 13.9417 | 0.0003 | ** |
| Error | 10 | 185569.7853 | 18556.9785 | . | . | - |
| Corrected Total | 17 | 1482678.4762 | . | . | . | - |
| ** - Significant at 1%, * - Significant  at 5%, NS - Non Significant | | | | | | |

Analysis of variance (ANOVA) for net returns of systems under different scenarios during year 2017-18

| **Source** | **DF** | **Type III SS** | **Mean Square** | **F Value** | **Pr > F** | **Significant** |
| --- | --- | --- | --- | --- | --- | --- |
| Replication | 2 | 5191.2536 | 2595.6268 | 0.0699 | 0.9330 | NS |
| Treatment | 5 | 2145803.4988 | 429160.6998 | 11.5542 | 0.0007 | ** |
| Error | 10 | 371432.3471 | 37143.2347 | . | . | - |
| Corrected Total | 17 | 2522427.0995 | . | . | . | - |
| ** - Significant at 1%, * - Significant  at 5%, NS - Non Significant | | | | | | |

**Irrigation water**

Analysis of variance (ANOVA) for applied irrigation (mm/ha) of rice under different scenarios during year 2016

| **Source** | **DF** | **Type III SS** | **Mean Square** | **F Value** | **Pr > F** | **Significant** |
| --- | --- | --- | --- | --- | --- | --- |
| Replication | 2 | 117882.3333 | 58941.1667 | 2.7404 | 0.1125 | NS |
| Treatment | 5 | 9136342.6250 | 1827268.5250 | 84.9566 | <.0001 | ** |
| Error | 10 | 215082.6667 | 21508.2667 | . | . | - |
| Corrected Total | 17 | 9469307.6250 | . | . | . | - |
| ** - Significant at 1%, * - Significant  at 5%, NS - Non Significant | | | | | | |

Analysis of variance (ANOVA) for applied irrigation (mm/ha) of rice under different scenarios during year 2017

| **Source** | **DF** | **Type III SS** | **Mean Square** | **F Value** | **Pr > F** | **Significant** |
| --- | --- | --- | --- | --- | --- | --- |
| Replication | 2 | 46370.0833 | 23185.0417 | 3.2280 | 0.0829 | NS |
| Treatment | 5 | 9249613.1250 | 1849922.6250 | 257.5600 | <.0001 | ** |
| Error | 10 | 71824.9167 | 7182.4917 | . | . | - |
| Corrected Total | 17 | 9367808.1250 | . | . | . | - |
| ** - Significant at 1%, * - Significant  at 5%, NS - Non Significant | | | | | | |

Analysis of variance (ANOVA) for applied irrigation (mm/ha) of wheat under different scenarios during year 2016-17 and 2017-18

| **Source** | **DF** | **Type III SS** | **Mean Square** | **F Value** | **Pr > F** | **Significant** |
| --- | --- | --- | --- | --- | --- | --- |
| Replication | 2 | 21455.4344 | 10727.7172 | 9.1315 | 0.0055 | ** |
| Treatment | 5 | 143939.2428 | 28787.8486 | 24.5043 | <.0001 | ** |
| Error | 10 | 11748.0656 | 1174.8066 | . | . | - |
| Corrected Total | 17 | 177142.7428 | . | . | . | - |
| ** - Significant at 1%, * - Significant  at 5%, NS - Non Significant | | | | | | |

Analysis of variance (ANOVA) for applied irrigation (mm/ha) of wheat under different scenarios during year 2017-18

| **Source** | **DF** | **Type III SS** | **Mean Square** | **F Value** | **Pr > F** | **Significant** |
| --- | --- | --- | --- | --- | --- | --- |
| Replication | 2 | 25672.1111 | 12836.0556 | 1.8520 | 0.2069 | NS |
| Treatment | 5 | 179217.1111 | 35843.4222 | 5.1714 | 0.0133 | * |
| Error | 10 | 69310.5556 | 6931.0556 | . | . | - |
| Corrected Total | 17 | 274199.7778 | . | . | . | - |
| ** - Significant at 1%, * - Significant  at 5%, NS - Non Significant | | | | | | |

Analysis of variance (ANOVA) for applied irrigation (mm/ha) in system under different scenarios during year 2016-17

| **Source** | **DF** | **Type III SS** | **Mean Square** | **F Value** | **Pr > F** | **Significant** |
| --- | --- | --- | --- | --- | --- | --- |
| Replication | 2 | 223323.0011 | 111661.5006 | 5.0130 | 0.0310 | * |
| Treatment | 5 | 10721618.9178 | 2144323.7836 | 96.2677 | <.0001 | ** |
| Error | 10 | 222745.9989 | 22274.5999 | . | . | - |
| Corrected Total | 17 | 11167687.9178 | . | . | . | - |
| ** - Significant at 1%, * - Significant  at 5%, NS - Non Significant | | | | | | |

Analysis of variance (ANOVA) for applied irrigation (mm/ha) in system under different scenarios during year 2017-18

| **Source** | **DF** | **Type III SS** | **Mean Square** | **F Value** | **Pr > F** | **Significant** |
| --- | --- | --- | --- | --- | --- | --- |
| Replication | 2 | 138105.3611 | 69052.6806 | 4.5136 | 0.0401 | * |
| Treatment | 5 | 10855146.5350 | 2171029.3070 | 141.9088 | <.0001 | ** |
| Error | 10 | 152987.6389 | 15298.7639 | . | . | - |
| Corrected Total | 17 | 11146239.5350 | . | . | . | - |
| ** - Significant at 1%, * - Significant  at 5%, NS - Non Significant | | | | | | |

**Irrigation water productivity**

Analysis of variance (ANOVA) for water productivity of rice/maize under different scenarios during year 2016-17

| **Source** | **DF** | **Type III SS** | **Mean Square** | **F Value** | **Pr > F** | **Significant** |
| --- | --- | --- | --- | --- | --- | --- |
| Replication | 2 | 0.8651 | 0.4326 | 0.7803 | 0.4843 | NS |
| Treatment | 5 | 251.4504 | 50.2901 | 90.7135 | <.0001 | ** |
| Error | 10 | 5.5438 | 0.5544 | . | . | - |
| Corrected Total | 17 | 257.8594 | . | . | . | - |
| ** - Significant at 1%, * - Significant  at 5%, NS - Non Significant | | | | | | |

Analysis of variance (ANOVA) for water productivity of rice/maize under different scenarios during year 2017-18

| **Source** | **DF** | **Type III SS** | **Mean Square** | **F Value** | **Pr > F** | **Significant** |
| --- | --- | --- | --- | --- | --- | --- |
| Replication | 2 | 0.0680 | 0.0340 | 0.5802 | 0.5776 | NS |
| Treatment | 5 | 263.6982 | 52.7396 | 900.6456 | <.0001 | ** |
| Error | 10 | 0.5856 | 0.0586 | . | . | - |
| Corrected Total | 17 | 264.3518 | . | . | . | - |
| ** - Significant at 1%, * - Significant  at 5%, NS - Non Significant | | | | | | |

Analysis of variance (ANOVA) for water productivity of wheat under different scenarios during year 2016-17

| **Source** | **DF** | **Type III SS** | **Mean Square** | **F Value** | **Pr > F** | **Significant** |
| --- | --- | --- | --- | --- | --- | --- |
| Replication | 2 | 1.6209 | 0.8105 | 9.3533 | 0.0051 | ** |
| Treatment | 5 | 16.3168 | 3.2634 | 37.6616 | <.0001 | ** |
| Error | 10 | 0.8665 | 0.0866 | . | . | - |
| Corrected Total | 17 | 18.8042 | . | . | . | - |
| ** - Significant at 1%, * - Significant  at 5%, NS - Non Significant | | | | | | |

Analysis of variance (ANOVA) for water productivity of wheat under different scenarios during year 2017-18

| **Source** | **DF** | **Type III SS** | **Mean Square** | **F Value** | **Pr > F** | **Significant** |
| --- | --- | --- | --- | --- | --- | --- |
| Replication | 2 | 0.4265 | 0.2133 | 1.9716 | 0.1897 | NS |
| Treatment | 5 | 8.7039 | 1.7408 | 16.0938 | 0.0002 | ** |
| Error | 10 | 1.0816 | 0.1082 | . | . | - |
| Corrected Total | 17 | 10.2120 | . | . | . | - |
| ** - Significant at 1%, * - Significant  at 5%, NS - Non Significant | | | | | | |

Analysis of variance (ANOVA) for water productivity of system under different scenarios during year 2016-17

| **Source** | **DF** | **Type III SS** | **Mean Square** | **F Value** | **Pr > F** | **Significant** |
| --- | --- | --- | --- | --- | --- | --- |
| Replication | 2 | 0.3186 | 0.1593 | 3.7207 | 0.0620 | NS |
| Treatment | 5 | 34.5350 | 6.9070 | 161.3274 | <.0001 | ** |
| Error | 10 | 0.4281 | 0.0428 | . | . | - |
| Corrected Total | 17 | 35.2817 | . | . | . | - |
| ** - Significant at 1%, * - Significant  at 5%, NS - Non Significant | | | | | | |

Analysis of variance (ANOVA) for water productivity of system under different scenarios during year 2017-18

| **Source** | **DF** | **Type III SS** | **Mean Square** | **F Value** | **Pr > F** | **Significant** |
| --- | --- | --- | --- | --- | --- | --- |
| Replication | 2 | 0.0311 | 0.0156 | 6.0835 | 0.0187 | * |
| Treatment | 5 | 33.7088 | 6.7418 | 2634.330 | <.0001 | ** |
| Error | 10 | 0.0256 | 0.0026 | . | . | - |
| Corrected Total | 17 | 33.7655 | . | . | . | - |
| ** - Significant at 1%, * - Significant  at 5%, NS - Non Significant | | | | | | |

**Energy input**

Analysis of variance (ANOVA) for energy input of rice under different scenarios during year 2016-17

| **Source** | **DF** | **Type III SS** | **Mean Square** | **F Value** | **Pr > F** | **Significant** |
| --- | --- | --- | --- | --- | --- | --- |
| Replication | 2 | 27596783.6338 | 13798391.8169 | 2.8328 | 0.1060 | NS |
| Treatment | 5 | 3028878493.2558 | 605775698.6512 | 124.3656 | <.0001 | ** |
| Error | 10 | 48709283.4046 | 4870928.3405 | . | . | - |
| Corrected Total | 17 | 3105184560.2942 | . | . | . | - |
| ** - Significant at 1%, * - Significant  at 5%, NS - Non Significant | | | | | | |

Analysis of variance (ANOVA) for energy input of rice under different scenarios during year 2017-18

| **Source** | **DF** | **Type III SS** | **Mean Square** | **F Value** | **Pr > F** | **Significant** |
| --- | --- | --- | --- | --- | --- | --- |
| Replication | 2 | 11042496.0274 | 5521248.0137 | 3.4392 | 0.0730 | NS |
| Treatment | 5 | 3048022701.2183 | 609604540.2437 | 379.7252 | <.0001 | ** |
| Error | 10 | 16053833.2170 | 1605383.3217 | . | . | - |
| Corrected Total | 17 | 3075119030.4628 | . | . | . | - |
| ** - Significant at 1%, * - Significant  at 5%, NS - Non Significant | | | | | | |

Analysis of variance (ANOVA) for energy input of wheat under different scenarios during year 2016-17

| **Source** | **DF** | **Type III SS** | **Mean Square** | **F Value** | **Pr > F** | **Significant** |
| --- | --- | --- | --- | --- | --- | --- |
| Replication | 2 | 11042496.0274 | 5521248.0137 | 3.4392 | 0.0730 | NS |
| Treatment | 5 | 3048022701.2183 | 609604540.2437 | 379.7252 | <.0001 | ** |
| Error | 10 | 16053833.2170 | 1605383.3217 | . | . | - |
| Corrected Total | 17 | 3075119030.4628 | . | . | . | - |
| ** - Significant at 1%, * - Significant  at 5%, NS - Non Significant | | | | | | |

Analysis of variance (ANOVA) for energy input of wheat under different scenarios during year 2017-18

| **Source** | **DF** | **Type III SS** | **Mean Square** | **F Value** | **Pr > F** | **Significant** |
| --- | --- | --- | --- | --- | --- | --- |
| Replication | 2 | 5308617.0319 | 2654308.5160 | 1.7665 | 0.2203 | NS |
| Treatment | 5 | 174267159.5353 | 34853431.9071 | 23.1953 | <.0001 | ** |
| Error | 10 | 15026075.7553 | 1502607.5755 | . | . | - |
| Corrected Total | 17 | 194601852.3225 | . | . | . | - |
| ** - Significant at 1%, * - Significant  at 5%, NS - Non Significant | | | | | | |

Analysis of variance (ANOVA) for energy input of system under different scenarios during year 2016-17

| **Source** | **DF** | **Type III SS** | **Mean Square** | **F Value** | **Pr > F** | **Significant** |
| --- | --- | --- | --- | --- | --- | --- |
| Replication | 2 | 50462462.3056 | 25231231.1528 | 4.8833 | 0.0331 | * |
| Treatment | 5 | 4148792969.1358 | 829758593.8272 | 160.5920 | <.0001 | ** |
| Error | 10 | 51668725.6274 | 5166872.5627 | . | . | - |
| Corrected Total | 17 | 4250924157.0688 | . | . | . | - |
| ** - Significant at 1%, * - Significant  at 5%, NS - Non Significant | | | | | | |

Analysis of variance (ANOVA) for energy input of system under different scenarios during year 2017-18

| **Source** | **DF** | **Type III SS** | **Mean Square** | **F Value** | **Pr > F** | **Significant** |
| --- | --- | --- | --- | --- | --- | --- |
| Replication | 2 | 30959913.0449 | 15479956.5225 | 4.6386 | 0.0376 | * |
| Treatment | 5 | 3901157963.8086 | 780231592.7617 | 233.7999 | <.0001 | ** |
| Error | 10 | 33371769.2997 | 3337176.9300 | . | . | - |
| Corrected Total | 17 | 3965489646.1533 | . | . | . | - |
| ** - Significant at 1%, * - Significant  at 5%, NS - Non Significant | | | | | | |

**Energy output**

Analysis of variance (ANOVA) for energy output of rice under different scenarios during year 2016-17

| **Source** | **DF** | **Type III SS** | **Mean Square** | **F Value** | **Pr > F** | **Significant** |
| --- | --- | --- | --- | --- | --- | --- |
| Replication | 2 | 217034217.7726 | 108517108.8863 | 0.7232 | 0.5089 | NS |
| Treatment | 5 | 4037177320.7092 | 807435464.1418 | 5.3814 | 0.0117 | * |
| Error | 10 | 1500427507.3159 | 150042750.7316 | . | . | - |
| Corrected Total | 17 | 5754639045.7977 | . | . | . | - |
| ** - Significant at 1%, * - Significant  at 5%, NS - Non Significant | | | | | | |

Analysis of variance (ANOVA) for energy output of rice under different scenarios during year 2017-18

| **Source** | **DF** | **Type III SS** | **Mean Square** | **F Value** | **Pr > F** | **Significant** |
| --- | --- | --- | --- | --- | --- | --- |
| Replication | 2 | 28470840.0979 | 14235420.0490 | 0.0997 | 0.9060 | NS |
| Treatment | 5 | 18027984261.8428 | 3605596852.3686 | 25.2502 | <.0001 | ** |
| Error | 10 | 1427946097.2647 | 142794609.7265 | . | . | - |
| Corrected Total | 17 | 19484401199.2054 | . | . | . | - |
| ** - Significant at 1%, * - Significant  at 5%, NS - Non Significant | | | | | | |

Analysis of variance (ANOVA) for energy output of wheat under different scenarios during year 2016-17

| **Source** | **DF** | **Type III SS** | **Mean Square** | **F Value** | **Pr > F** | **Significant** |
| --- | --- | --- | --- | --- | --- | --- |
| Replication | 2 | 56352661.8122 | 28176330.9061 | 0.3258 | 0.7293 | NS |
| Treatment | 5 | 1748843472.5154 | 349768694.5031 | 4.0449 | 0.0287 | * |
| Error | 10 | 864718088.6680 | 86471808.8668 | . | . | - |
| Corrected Total | 17 | 2669914222.9956 | . | . | . | - |
| ** - Significant at 1%, * - Significant  at 5%, NS - Non Significant | | | | | | |

Analysis of variance (ANOVA) for energy output of wheat under different scenarios during year 2017-18

| **Source** | **DF** | **Type III SS** | **Mean Square** | **F Value** | **Pr > F** | **Significant** |
| --- | --- | --- | --- | --- | --- | --- |
| Replication | 2 | 53720369.9170 | 26860184.9585 | 0.3774 | 0.6950 | NS |
| Treatment | 5 | 608286381.1471 | 121657276.2294 | 1.7093 | 0.2200 | NS |
| Error | 10 | 711757435.7170 | 71175743.5717 | . | . | - |
| Corrected Total | 17 | 1373764186.7811 | . | . | . | - |
| ** - Significant at 1%, * - Significant  at 5%, NS - Non Significant | | | | | | |

Analysis of variance (ANOVA) for energy output of system under different scenarios during year 2016-17

| **Source** | **DF** | **Type III SS** | **Mean Square** | **F Value** | **Pr > F** | **Significant** |
| --- | --- | --- | --- | --- | --- | --- |
| Replication | 2 | 72949880.3774 | 36474940.1887 | 0.1175 | 0.8904 | NS |
| Treatment | 5 | 667541149.4599 | 133508229.8920 | 0.4300 | 0.8179 | NS |
| Error | 10 | 3104489016.3004 | 310448901.6300 | . | . | - |
| Corrected Total | 17 | 3844980046.1377 | . | . | . | - |
| ** - Significant at 1%, * - Significant  at 5%, NS - Non Significant | | | | | | |

Analysis of variance (ANOVA) for energy output of system under different scenarios during year 2017-18

| **Source** | **DF** | **Type III SS** | **Mean Square** | **F Value** | **Pr > F** | **Significant** |
| --- | --- | --- | --- | --- | --- | --- |
| Replication | 2 | 12897259.9957 | 6448629.9978 | 0.0225 | 0.9778 | NS |
| Treatment | 5 | 22054485901.3797 | 4410897180.2759 | 15.4079 | 0.0002 | ** |
| Error | 10 | 2862755355.7942 | 286275535.5794 | . | . | - |
| Corrected Total | 17 | 24930138517.1696 | . | . | . | - |
| ** - Significant at 1%, * - Significant  at 5%, NS - Non Significant | | | | | | |

**Energy use efficiency**

Analysis of variance (ANOVA) for energy use efficiency of rice under different scenarios during year 2016-17

| **Source** | **DF** | **Type III SS** | **Mean Square** | **F Value** | **Pr > F** | **Significant** |
| --- | --- | --- | --- | --- | --- | --- |
| Replication | 2 | 1.1155 | 0.5578 | 4.3977 | 0.0426 | * |
| Treatment | 5 | 301.8417 | 60.3683 | 475.9851 | <.0001 | ** |
| Error | 10 | 1.2683 | 0.1268 | . | . | - |
| Corrected Total | 17 | 304.2255 | . | . | . | - |
| ** - Significant at 1%, * - Significant  at 5%, NS - Non Significant | | | | | | |

Analysis of variance (ANOVA) for energy use efficiency of rice under different scenarios during year 2017-18

| **Source** | **DF** | **Type III SS** | **Mean Square** | **F Value** | **Pr > F** | **Significant** |
| --- | --- | --- | --- | --- | --- | --- |
| Replication | 2 | 0.2686 | 0.1343 | 0.4092 | 0.6748 | NS |
| Treatment | 5 | 792.8070 | 158.5614 | 483.2021 | <.0001 | ** |
| Error | 10 | 3.2815 | 0.3281 | . | . | - |
| Corrected Total | 17 | 796.3570 | . | . | . | - |
| ** - Significant at 1%, * - Significant  at 5%, NS - Non Significant | | | | | | |

Analysis of variance (ANOVA) for energy use efficiency of wheat under different scenarios during year 2016-17

| **Source** | **DF** | **Type III SS** | **Mean Square** | **F Value** | **Pr > F** | **Significant** |
| --- | --- | --- | --- | --- | --- | --- |
| Replication | 2 | 1.5112 | 0.7556 | 2.6077 | 0.1226 | NS |
| Treatment | 5 | 37.2693 | 7.4539 | 25.7240 | <.0001 | ** |
| Error | 10 | 2.8976 | 0.2898 | . | . | - |
| Corrected Total | 17 | 41.6782 | . | . | . | - |
| ** - Significant at 1%, * - Significant  at 5%, NS - Non Significant | | | | | | |

Analysis of variance (ANOVA) for energy use efficiency of wheat under different scenarios during year 2017-18

| **Source** | **DF** | **Type III SS** | **Mean Square** | **F Value** | **Pr > F** | **Significant** |
| --- | --- | --- | --- | --- | --- | --- |
| Replication | 2 | 1.1003 | 0.5501 | 1.7601 | 0.2214 | NS |
| Treatment | 5 | 35.8722 | 7.1744 | 22.9536 | <.0001 | ** |
| Error | 10 | 3.1256 | 0.3126 | . | . | - |
| Corrected Total | 17 | 40.0980 | . | . | . | - |
| ** - Significant at 1%, * - Significant  at 5%, NS - Non Significant | | | | | | |

Analysis of variance (ANOVA) for energy use efficiency of system under different scenarios during year 2016-17

| **Source** | **DF** | **Type III SS** | **Mean Square** | **F Value** | **Pr > F** | **Significant** |
| --- | --- | --- | --- | --- | --- | --- |
| Replication | 2 | 0.6935 | 0.3467 | 2.7224 | 0.1138 | NS |
| Treatment | 5 | 92.2043 | 18.4409 | 144.7912 | <.0001 | ** |
| Error | 10 | 1.2736 | 0.1274 | . | . | - |
| Corrected Total | 17 | 94.1714 | . | . | . | - |
| ** - Significant at 1%, * - Significant  at 5%, NS - Non Significant | | | | | | |

Analysis of variance (ANOVA) for energy use efficiency of system under different scenarios during year 2017-18

| **Source** | **DF** | **Type III SS** | **Mean Square** | **F Value** | **Pr > F** | **Significant** |
| --- | --- | --- | --- | --- | --- | --- |
| Replication | 2 | 0.4466 | 0.2233 | 1.8721 | 0.2039 | NS |
| Treatment | 5 | 175.9327 | 35.1865 | 295.0026 | <.0001 | ** |
| Error | 10 | 1.1928 | 0.1193 | . | . | - |
| Corrected Total | 17 | 177.5720 | . | . | . | - |
| ** - Significant at 1%, * - Significant  at 5%, NS - Non Significant | | | | | | |

**Energy productivity**

Analysis of variance (ANOVA) for energy productivity of rice under different scenarios during year 2016

| **Source** | **DF** | **Type III SS** | **Mean Square** | **F Value** | **Pr > F** | **Significant** |
| --- | --- | --- | --- | --- | --- | --- |
| Replication | 2 | 0.0015 | 0.0007 | 1.6138 | 0.2469 | NS |
| Treatment | 5 | 0.6029 | 0.1206 | 264.9746 | <.0001 | ** |
| Error | 10 | 0.0046 | 0.0005 | . | . | - |
| Corrected Total | 17 | 0.6089 | . | . | . | - |
| ** - Significant at 1%, * - Significant  at 5%, NS - Non Significant | | | | | | |

Analysis of variance (ANOVA) for energy productivity of rice under different scenarios during year 2017

| **Source** | **DF** | **Type III SS** | **Mean Square** | **F Value** | **Pr > F** | **Significant** |
| --- | --- | --- | --- | --- | --- | --- |
| Replication | 2 | 0.0008 | 0.0004 | 0.7431 | 0.5001 | NS |
| Treatment | 5 | 0.6707 | 0.1341 | 260.9736 | <.0001 | ** |
| Error | 10 | 0.0051 | 0.0005 | . | . | - |
| Corrected Total | 17 | 0.6766 | . | . | . | - |
| ** - Significant at 1%, * - Significant  at 5%, NS - Non Significant | | | | | | |

Analysis of variance (ANOVA) for energy productivity of wheat under different scenarios during year 2016-17

| **Source** | **DF** | **Type III SS** | **Mean Square** | **F Value** | **Pr > F** | **Significant** |
| --- | --- | --- | --- | --- | --- | --- |
| Replication | 2 | 0.0046 | 0.0023 | 2.8988 | 0.1016 | NS |
| Treatment | 5 | 0.0761 | 0.0152 | 18.9825 | 0.0001 | ** |
| Error | 10 | 0.0080 | 0.0008 | . | . | - |
| Corrected Total | 17 | 0.0887 | . | . | . | - |
| ** - Significant at 1%, * - Significant  at 5%, NS - Non Significant | | | | | | |

Analysis of variance (ANOVA) for energy productivity of wheat under different scenarios during year 2017-18

| **Source** | **DF** | **Type III SS** | **Mean Square** | **F Value** | **Pr > F** | **Significant** |
| --- | --- | --- | --- | --- | --- | --- |
| Replication | 2 | 0.0008 | 0.0004 | 1.0675 | 0.3800 | NS |
| Treatment | 5 | 0.0708 | 0.0142 | 39.1158 | <.0001 | ** |
| Error | 10 | 0.0036 | 0.0004 | . | . | - |
| Corrected Total | 17 | 0.0752 | . | . | . | - |
| ** - Significant at 1%, * - Significant  at 5%, NS - Non Significant | | | | | | |

Analysis of variance (ANOVA) for energy productivity of system under different scenarios during year 2016-17

| **Source** | **DF** | **Type III SS** | **Mean Square** | **F Value** | **Pr > F** | **Significant** |
| --- | --- | --- | --- | --- | --- | --- |
| Replication | 2 | 0.0016 | 0.0008 | 3.3821 | 0.0755 | NS |
| Treatment | 5 | 0.2001 | 0.0400 | 171.7248 | <.0001 | ** |
| Error | 10 | 0.0023 | 0.0002 | . | . | - |
| Corrected Total | 17 | 0.2041 | . | . | . | - |
| ** - Significant at 1%, * - Significant  at 5%, NS - Non Significant | | | | | | |

Analysis of variance (ANOVA) for energy productivity of system under different scenarios during year 2017-18

| **Source** | **DF** | **Type III SS** | **Mean Square** | **F Value** | **Pr > F** | **Significant** |
| --- | --- | --- | --- | --- | --- | --- |
| Replication | 2 | 0.0003 | 0.0001 | 0.8048 | 0.4741 | NS |
| Treatment | 5 | 0.2295 | 0.0459 | 248.2040 | <.0001 | ** |
| Error | 10 | 0.0018 | 0.0002 | . | . | - |
| Corrected Total | 17 | 0.2317 | . | . | . | - |
| ** - Significant at 1%, * - Significant  at 5%, NS - Non Significant | | | | | | |
